# Supplementary material for: Structure sensitivity in gas sorption and conversion on metal-organic frameworks
Source: Nat Commun. 2023 Jan 9;14:129. doi: 10.1038/s41467-022-35762-9 (PMC9829675; doi:10.1038/s41467-022-35762-9)
Supplement: Supplementary file 1 — Supplementary Information [file 41467_2022_35762_MOESM1_ESM.pdf]

# Supporting Information

**Structure Sensitivity in Gas Sorption and Conversion on Metal-Organic Frameworks**

Guusje Delen *et al.*

**This PDF file includes:**

Supplementary Materials and Methods

Supplementary Text

Supplementary Figures 1 to 40

Supplementary Tables 1 to 4

Supplementary References

## Supplementary Methods

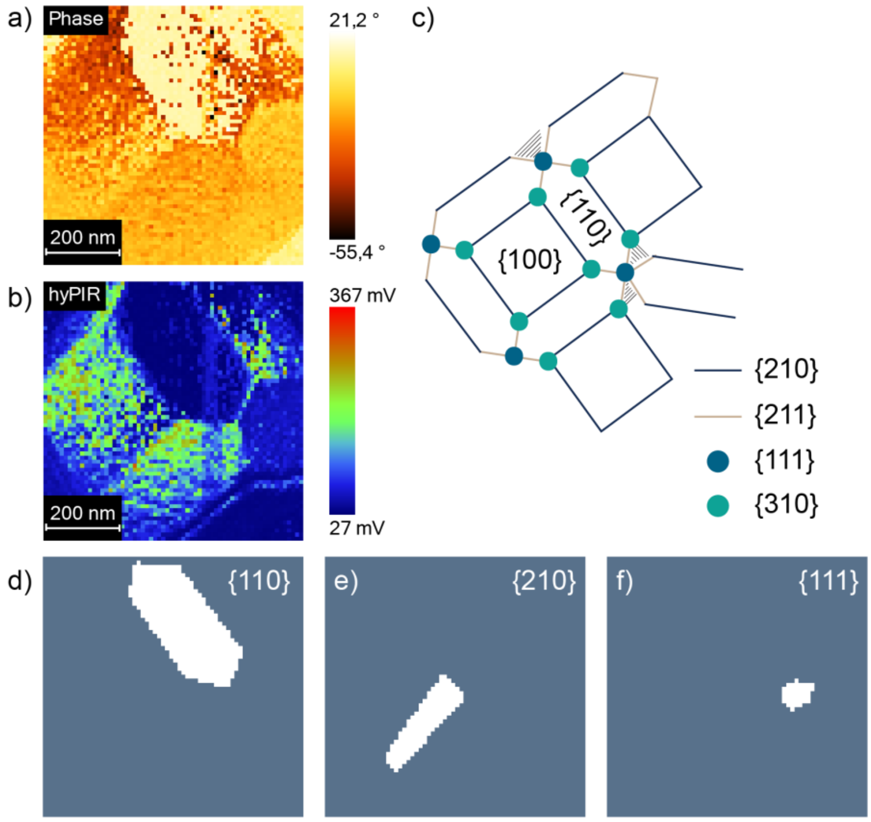


**Supplementary Figure 1.**

Experimental approach for crystal-plane specific analysis. a) Highly defined ZIF-8 crystals provide facile identification of crystal planes in b) hyperspectral images at varying formaldehyde pressure. c) Measurement-specific crystal plane maps were drawn for the construction of masks. d, e, and f) gives examples of a mask used for a facet, edge, and corner, respectively. Spectra of individual masks were analyzed for outlier behavior before averaging plane-dependent masked spectra to improve the S/N ratio.

## Density Functional Theory calculations

Surface models

Using density functional theory (DFT) we studied the nature of surface terminations of three ZIF-8 orientations, namely {100}, {110} and {310} crystal planes. It is crucial to realize that for each of the {100}, {110} and {310} ZIF-8 planes, different surfaces can be constructed by varying the slicing height as illustrated in Supplementary Figure 2. For both {100} and {100}, and the {310} surface, two and three surface terminations are possible, respectively, which differ in the density of Zn atoms per surface area as well as in the number of Zn-N bonds cleaved, giving rise to various under-coordinated sites. In our model, we assumed that surface terminations were always neutral as was done in the work of Weng and Schmidt, i.e., charged surface terminations are not considered as they require diffusive double layer of ions from solution to maintain overall charge neutrality of the system.[3] It has been shown that surface Zn-sites can be unsaturated or capped by various terminating groups, such as hydroxyl, carbonate, and monodentate 2-methylimidazole (Mim), depending on the system conditions.[3,4] The theoretical work of Weng and Schmidt suggests that the most plausible surface terminations are primarily imidazole-related species.[3] Thus, in all our models we saturated dangling Zn-N bonds with protonated 2-methylimidazole (Hmim) with the aim to elucidate general structural characteristics of various ZIF-8 facets, independently of system conditions. For comparison, we also considered zero coverage of surfaces.

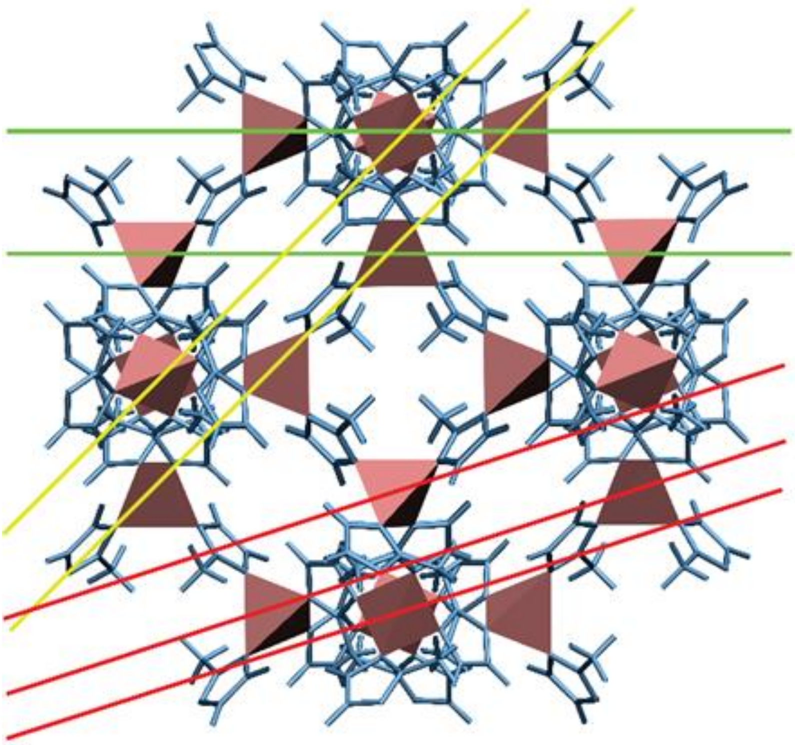

**Supplementary Figure 2.**
Different heights of cleavage along the {100}, {110} and {310} planes visualized by green, yellow, and red lines, respectively.

In the next step, adsorption of formaldehyde was studied by adsorbing one furfuryl alcohol molecule on each side of the clean surface.

To create a defective surface models, we replaced one terminal Mim with a pyrrole. A cleavage of the surfaces lead to a large number of surface sites, and it is impossible to systematically examine the stability of the pyrrole linker with respect to all possible positions of a defect site. Instead, by analyzing the local structure of each surface site we identified a set of surface motifs, shown in Supplementary Figure 3, which serve as universal descriptors of the surface terminations. In the next step, we then introduced a defect site by replacing a Mim linker with pyrrole on each of the surface motifs. We did so for both cleavages of {100} and {110} facets and for the surface sites on the most stable cleavage of the {310} plane. The surface was subsequently re-optimized. The adsorption of formaldehyde followed by the adsorption of water and second formaldehyde was also modelled in the same manner as for pristine surfaces.

Computational details

DFT calculations were conducted using version 6.1. of CP2K software, within Gaussian and plane wave (GPW) approach.[5] GTH pseudopotentials combined with TZVP-MOLOPT basis set and plane-wave density cutoff 700 Ry were employed. The target convergence of SCF cycle was set to $10^{-6}$. All geometries were optimized using PBE exchange-correlation functional with a semi-empirical Grimme D3 correction.[6,7] Initially, the bulk structure together with the unit cell was optimized and after that unit cell dimensions were kept fixed. The optimized unit cell dimensions were 17.069 Å in each direction, i.e. a cubic representation of the bulk ZIF-8 was used. Subsequently, all possible {100}, {110} and {310} surface terminations were constructed, and the structure was re-optimized.

**Supplementary Table 1.**

The unit cell parameters, which were used for the models of various surface orientations and cleavages. The naming system corresponds to the one presented in Supplementary Figure 3.

| **surface** | **a [Å]** | **b [Å]** | **c [Å]** | **α [°]** | **β [°]** | **γ [°]** |
|---|---|---|---|---|---|---|
| **100-0** | 37.069 | 17.069 | 17.069 | 90.00 | 90.00 | 90.00 |
| **100-1** | 37.069 | 17.069 | 17.069 | 90.00 | 90.00 | 90.00 |
| **110-0** | 45.139 | 24.139 | 17.069 | 90.00 | 90.00 | 90.00 |
| **110-1** | 45.139 | 24.139 | 17.069 | 90.00 | 90.00 | 90.00 |
| **310-0** | 53.977 | 35.069 | 17.069 | 90.00 | 90.00 | 71.57 |
| **310-1** | 53.977 | 40.069 | 17.069 | 90.00 | 90.00 | 71.57 |
| **310-2** | 53.977 | 45.069 | 17.069 | 90.00 | 90.00 | 71.57 |

The unit cell parameters of each slab are summarized in Table S1. Each of the surfaces was approximated by symmetric two-dimensional periodic slab models separated from each other in a perpendicular direction by a layer of vacuum at least 15 Å thick. To avoid interaction of the two surfaces within the same slab, the convergence of the slab thickness with respect to surface energy was tested. Based on the results, the slab depth of at least five Zn layers was chosen for each of the models. During the optimization, the middle Zn layers were fixed, while at least the top two Zn layers were allowed to relax. The thermodynamically most stable surface was the one with minimal surface energy, which was computed for a saturated surface as:

$$\gamma^{sat} = \frac{1}{2A}\left(E_{slab} - NE_{Zn(Mim)_2} - nE_{Hmim}\right)$$

and for an unsaturated (clean) surface as:

$$\gamma^{unsat} = \frac{1}{2A}\left(E_{slab} - NE_{Zn(Mim)_2}\right)$$

$E_{slab}$ is the energy of the symmetric slab with a total surface area of *2A* and *N* and $E_{Zn(Mim)_2}$ are the number of the elementary building blocks in the slab and corresponding energy computed from the bulk structure, respectively, and *n* and $E_{Hmim}$ are the number of terminal protonated imidazole linkers (Hmim) and corresponding energy. Within our model, we have used $\gamma^{sat}$ to determine preferred termination of pristine {100}, {110} and {310} facets.

In defective models, the concentration of the pyrrole was always fixed to one defect per surface (i.e. two pyrroles per unit cell). Due to varying unit cell size with respect to the surface model it was not possible to compare surface energies in order to compare their stability. Instead, reaction energy was used:

$$\boldsymbol{dE^{pyrrole} = \frac{1}{2}\left(E_{slab-pyrrole} + 2E_{Hmim} - E_{slab} - 2E_{pyrrole}\right)}$$

Where $\boldsymbol{E_{slab-pyrrole}}$ is the energy of a defective slab and $\boldsymbol{E_{pyrrole}}$ and $\boldsymbol{E_{mim}}$ are the gas phase energies of neutral pyrrole and neutral methyl-imidazole in the gas-phase. The factor ½ originates from the symmetry of the slab.

The adsorption of formaldehyde (FA) was similarly expressed in terms of reaction energy required to replace one Hmim per surface with the reactant molecule:

$$\boldsymbol{dE^{FA} = \frac{1}{2}(E_{slab-FA} + 2E_{Hmim} - E_{slab} - 2E_{FA})}$$

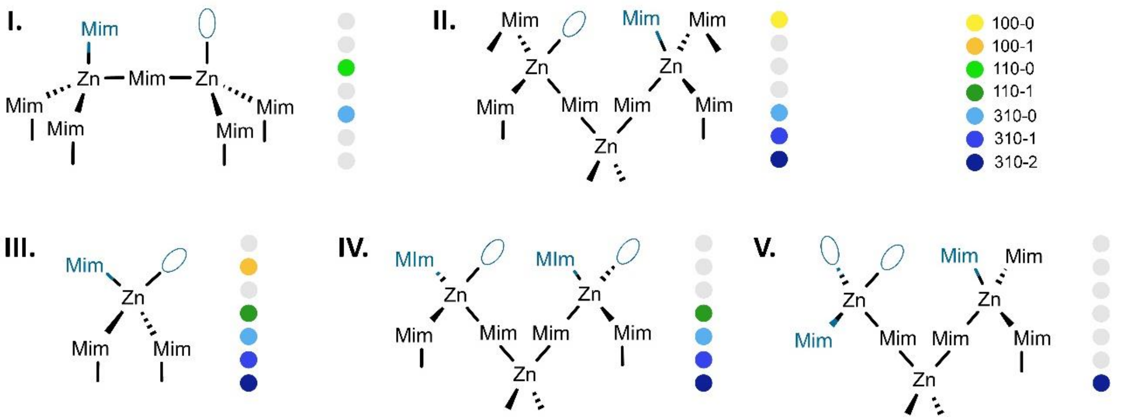


**Supplementary Figure 1.**

where $\boldsymbol{E_{slab-FA}}$ is the energy of the original surface and $\boldsymbol{E_{FA}}$ is the energy of the adsorbent in the gas-phase. The goal of an introduction of the reaction energy is to allow a straightforward comparison of the stability of various adsorbates on both a pristine and a defective surface.

A schematic representation of various surface motifs. The colored scheme shows for which surface orientation and a cleavage height is the given surface motif present, e.g. motif V can be found only on 310-2 surface (Supplementary Figure 2). In the saturated surface model are empty terminal sites, visualized by a blue ellipse, filled with a capping Hmim.

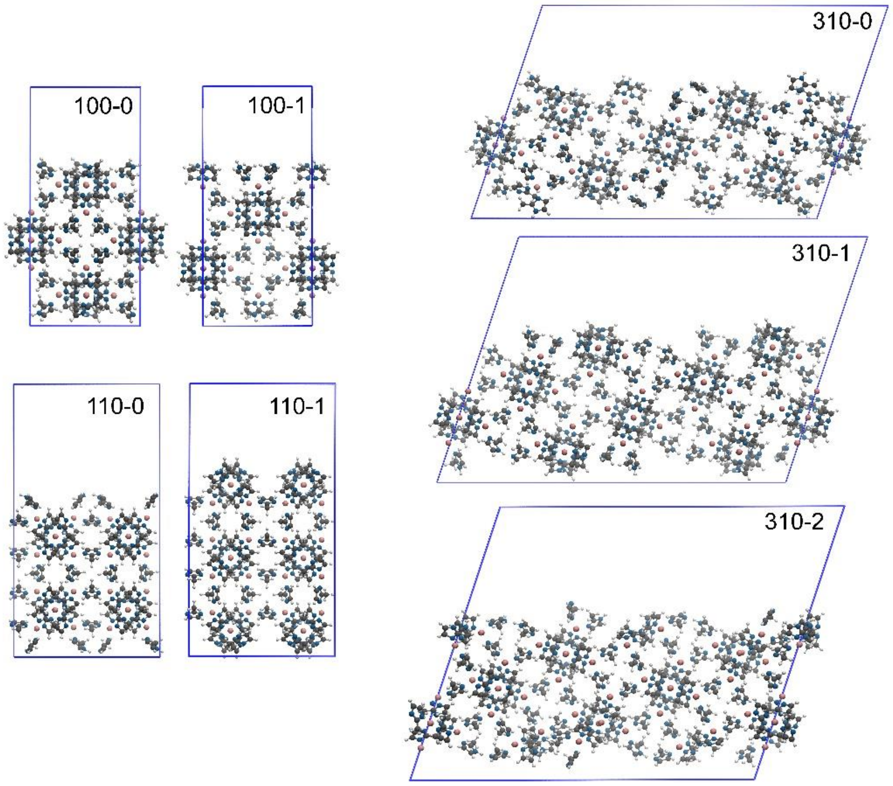


**Supplementary Figure 4.**

Optimized surface models of various cleavages of {100}, {110} and {310} planes. Undercoordinated terminal sites were saturated with Hmim.

**The structure and thermodynamic stability of selected ZIF-8 facets**

Pristine and defective surface models

First, we constructed a set of pristine surface models, which differ in their surface orientation ({100}, {110}, {310}) and cleavage height. The optimized structures are shown in Supplementary Figure 4 and calculated surface energies are shown in Table S2. Consistent with AFM observations (Supplementary Figure 10), we find that the {110} surface is the most stable ($\Upsilon^{sat}$ = -4.91 meV/Å$^2$), followed by {100} surface ($\Upsilon^{sat}$ = -3.21 meV/Å$^2$) and {310} which is the least stable ($\Upsilon^{sat}$ = -2.67 meV/Å$^2$). The higher energy cuts display surface energies between -1.05 meV/Å$^2$ (310-2, Figure 3) and 3.45 meV/Å$^2$ (100-1, Figure 3). Each of the studied surfaces is terminated with different surface motifs visualized in the Supplementary Figure 3. We find no apparent correlation between the number of terminal linkers or density of surface sites and surface energy. The reduction of the surface energy to negative values is the result of the exothermic adsorption of Hmim on the terminal Zn atoms and have been observed for other systems including zeolites.[8–10] To eliminate the influence of adsorbates on the relative stability of the surfaces, we extended our study about the models of empty surfaces with unsaturated terminal Zn nodes (Table S2). We find that the saturation of the surface with Hmim has no effect on the ordering of different surface orientations, but it alters the ordering of different cleavage heights of the same surface orientation. We note that during the geometry optimization of unsaturated surfaces a significant surface reconstruction occurred during which terminal imidazole linkers migrated from less to the most unsaturated surface sites. These observations are in line with the computational work of Weng and Schmidt, who showed that a {110} surface with unsaturated terminal Zn atoms is almost never stable.[3] Based on these results, we have decided to proceed with the simulations of saturated surfaces only.

**Supplementary Table 2.**

Surface energies for different cleavages of {100}, {110} and {310} surface orientations. Two models were considered: an empty surface with unsaturated terminal Zn nodes and a saturated surface, in which are Zn atoms capped with Hmim ligands. The surfaces were characterized in terms of the surface area, concentration of terminal Zn atoms and a type of surface sites motifs present upon cleavage of the surface. The most stable terminations are highlighted in bold.

| **surface** | **cleavage** | **A [Å$^2$]** | $\gamma^{unsat}$ **[mev/Å$^2$]** | $\gamma^{sat}$ **[mev/Å$^2$]** | **[#Zn/u.c]** | **surface motifs** |
|---|---|---|---|---|---|---|
| 100 | -0 | 291.35 | 13.53 | **-3.21** | 4 | II |
| 100 | -1 | 291.35 | **12.34** | 3.45 | 2 | III |
| 110 | -0 | 412.03 | **10.11** | 0.62 | 4 | I |
| 110 | -1 | 412.03 | 16.50 | **-4.91** | 4 | III, IV |
| 310 | -0 | 921.33 | **13.16** | 2.08 | 10 | I, II, III |
| 310 | -1 | 921.33 | 15.03 | **-2.67** | 10 | II, III, IV |
| 310 | -2 | 921.33 | 15.48 | -1.05 | 8 | II, III, V |

The introduction of defects is modelled by replacing one terminal Mim with a pyrrole linker. A pyrrole defect can be introduced on the surface by replacing Mim linker of one of surface motifs shown in Supplementary Figure 3. We find that the reaction will preferentially occur on the motif III of high energy plane of {100} surface with the reaction energy 10.2 kJ/mol, followed by {310} and high energy plane of {110} surface with reaction energies 17.22 kJ/mol and 28.5 kJ/mol. If we assume, that the incorporation of defects will occur on high-energy planes of both {100} and {110} surfaces to the same extent, then the difference in the surface energy between the planes will decrease. This can lead to the reconstruction of the surface and a change in {100} and {110} planes ratio. Thermodynamically least favorable is the pyrrole introduction on low energy planes of both {100} and {110} facets as well as on other motifs of {310} surface with reaction energies between 37.8 kJ/mol and 54.6 kJ/mol (Table S3).

**Supplementary Table 3.**

Reaction energies corresponding to the introduction of a pyrrole defect on the surface of {100}, {110} and {310} planes. The position of the defect is indicated by the type of surface motif (Supplementary Figure 3) at which the Mim linker was replaced. Note that for the 110-1 surface the value is the same for surface motif III and IV since motif III can be formed from IV (see Supplementary Figure 3).

<table>
<tr><th>surface</th><th>cleavage</th><th>defect site</th><th>$dE^{pyrrole}$ [kJ/mol]</th></tr>
<tr><td>100</td><td>-0</td><td>II</td><td>52.3</td></tr>
<tr><td>100</td><td>-1</td><td>III</td><td>10.2</td></tr>
<tr><td>110</td><td>-0</td><td>I</td><td>28.5</td></tr>
<tr><td>110</td><td>-1</td><td>III</td><td rowspan="2">48.7</td></tr>
<tr><td>110</td><td>-1</td><td>IV</td></tr>
<tr><td>310</td><td>-1</td><td>II</td><td>54.6</td></tr>
<tr><td>310</td><td>-1</td><td>III</td><td>17.2</td></tr>
<tr><td>310</td><td>-1</td><td>IV</td><td>37.8</td></tr>
</table>

Formaldehyde adsorption

We undertook a systematic investigation of the formaldehyde (FA) adsorption on the possible surface motifs for the {100}, {110}, and {310} planes. While for {100} and {110} planes we systematically test FA adsorption on both low and high energy planes, for {310} corners we assume that only the most stable plane will be present due to its low surface area on the crystals. Our results are summarized in Supplementary Figure 5. We find that at 0 K a coordination of FA to the Zn atom is always less stable than a coordination of Hmim, i.e., the process is endothermic. The reaction requires the least amount of energy on the {310} plane with (25.3 kJ/mol) and on the high-energy plane of {100} surface (26.8 kJ/mol). The FA adsorption on the most stable {100} and {110} surfaces is less favorable with reaction energy of 74.1 and 57.7 kJ/mol, i.e. we predict formaldehyde sorption to occur in the following order: {310} > {110} > {100}, which coincides with experimental observations. The analysis of the stable structures revealed that FA coordination to the Zn atom is always accompanied by formation of a covalent C-N bond with a neighboring terminal Mim linker. In the most stable state – the FA adsorption on the isolated site III of {310} surface or of high energy plane of {100} –the terminal linker will be completely decoordinated from the Zn atom (Figure 3) and will produce an undercoordinated Zn atom. The same complex is unstable on the {100} facet with a reaction energy of 139.7 kJ/mol due to the presence of a neighboring terminal Zn atom leading to the formation of more stable motif IV. The complexes with one or three terminal groups (III-V) are always less reactive with reaction energies between 47.1 kJ/mol (motif I, {110}) and 119.0 kJ/mol (motif V, {310}). From this we conclude that the reactivity of the surface site is not determined by a number of terminal groups, but rather by a coordination number of the Zn atom and local environment.

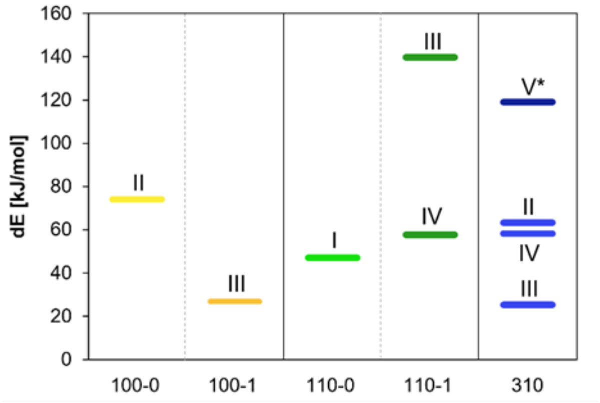


**Supplementary Figure 5.**

Reaction energies of formaldehyde (FA) upon adsorption on different surface motifs of {100}, {110} and {310} planes. Possible surface motifs are schematically shown in Supplementary Figure 3 and are indicated by roman numbers. For {100} and (110), both low and high energy planes were considered, while for the {310} surface we consider only the most stable cleavage (which still possesses multiple possible surface motifs). For completeness we also explored FA adsorption on the site V, which can be found only on a less stable (310-2) cut.

Next, we have explored the change in reaction energies of FA adsorption with respect to the incorporation of the defect (Supplementary Figure 6). In line with observations for the pristine surfaces, we find that the replacement of Hmim capping linker with FA is an endothermic reaction. Overall, FA binding on the low energy planes is the most favorable on {310} with reaction energy of 1.5 kJ/mol, followed by {110} and {100} with reaction energies 88.9 kJ/mol and 97.1 kJ/mol. When FA coordinates to the defective surface, the same $O_{FA}$-$Zn_{ZIF}$ and $C_{FA}$-$N_{ZIF}$ adducts as in the pristine case are formed, but the linker will be no longer coordinated to the Zn atom, since pyrrole's only N atom is bound to FA (Figure 5). This will lead to the formation of undercoordinated Zn sites displaying Lewis acidity which we propose to be responsible for the conversion of formaldehyde on defective ZIF-8 surfaces.[11,12]

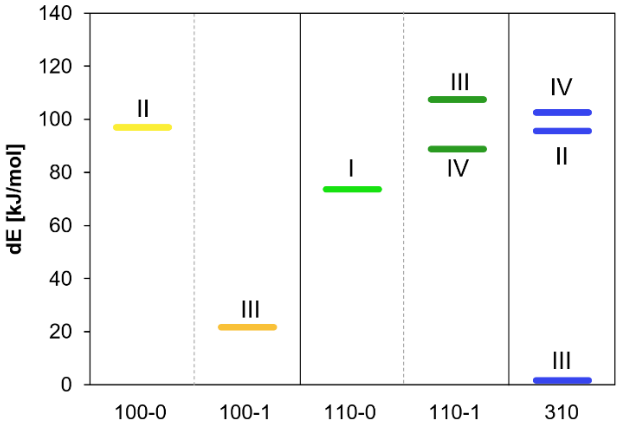


**Supplementary Figure 6.**

Reaction energies of the formaldehyde (FA) upon adsorption to different defective surface sites of {100}, {110} and {310} facets. Possible surface motifs are schematically shown in Supplementary Figure 3 and are indicated by roman numbers. For {100} and {110}, both low and high energy planes were considered, while for the {310} surface we consider only the most stable termination.

## Supplementary Results & Discussion

Structure sensitivity in heterogeneous catalysis

The performance of a functional material is often determined by only a small percentage of its surface sites and their atomic configuration. This phenomenon, known as structure sensitivity, describes the relationship between exposed crystal surfaces and rate of conversion and is well known in the field of heterogeneous catalysis.[13–15] This phenomenon occurs when distinct surface sites possess a different reactivity.[16] Changes in dispersion between surface sites thus results in a change in overall activity.[17]

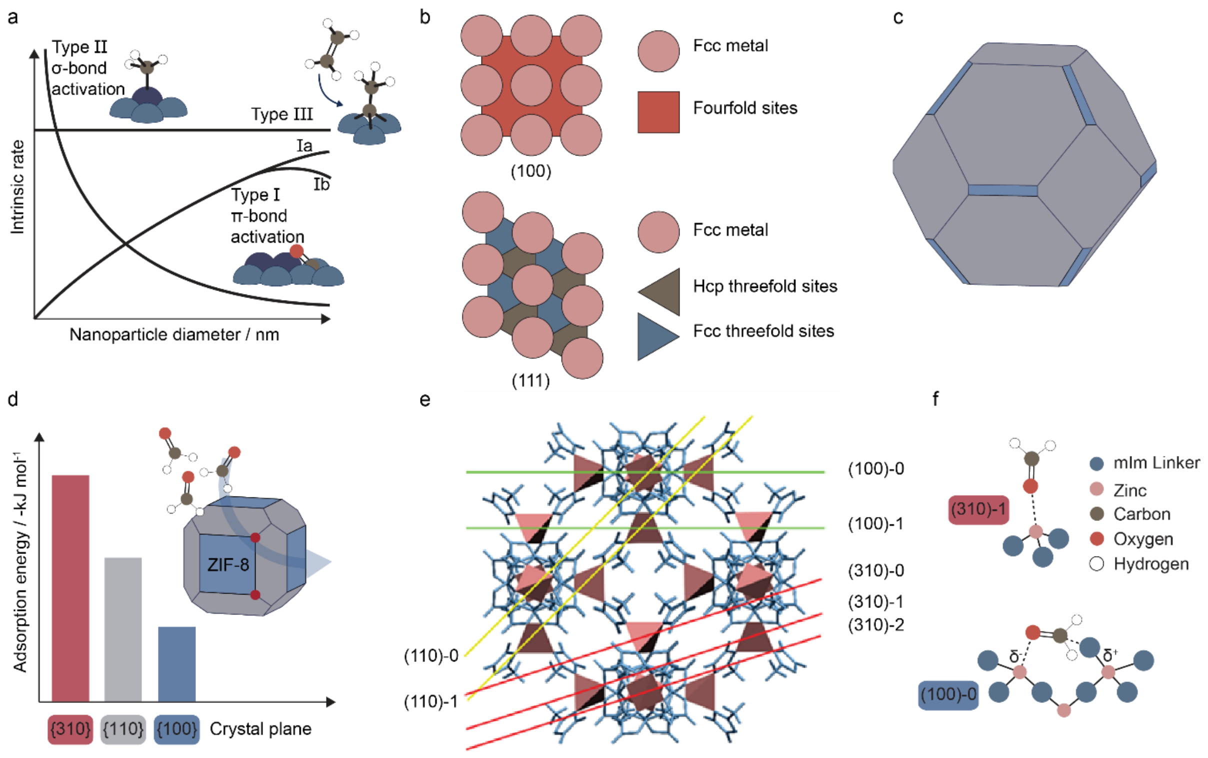


**Supplementary Figure 7.**

Schematic describing structure sensitivity in supported nanoparticles (NPs), and in porous functional materials. a) Three general types of structure sensitivity are recognized for supported NPs, where a relationship between intrinsic conversion rate and NP size is found based on which type of bond (sigma/pi) needs to be activated during the rate determining step. Adapted from [13] b) Different types of surface sites/ensembles are required for σ/π which can be found on different crystal planes. c) The dispersion of these crystal planes depends on the size of the metal NPs. d) Structure sensitivity for porous functional materials, e.g. ZIF-8, describes the crystal plane-dependent sorption and conversion energies of adsorbates. Adapted from [13,18]. e) For each crystal plane, multiple cuts can be made resulting in crystal plane conformations with alternate surface and adsorption energies. Two examples of

thermodynamically favored crystal plane conformations of ZIF-8 {310} and {100} planes, and their interaction with formaldehyde, are shown in (f).

Structure sensitivity is often described for supported metal nanoparticle catalysts, where the variation of metal nanoparticle size results in the exposure of a different fraction of active surface sites, leading to size-dependent performance (Supplementary Figure 7a-c).[19] Different types of structure sensitivity apply to specific chemical reactions. For example, in type II structure sensitivity, conversion is limited by the rate of π-bond activation which specifically occurs over highly active step-edge or kink sites. [20] These sites require ensembles of atoms and their fraction increases with NP size, up to a material-dependent maximum. Similarly, type I structure sensitivity describes the decrease in intrinsic rate with NP diameter due to a loss of isolated unsaturated sites for σ-bond activation.[16] Furthermore, for other chemical reactions this size-dependency is absent leading to the concept of structure insensitivity (type III).[21]

While for dense, non-porous metal nanoparticles it is relatively easy to distinguish between bulk atoms and the wide variety of distinct surface atoms, this distinction is more difficult for porous functional materials, such as zeolites and metal-organic frameworks (MOFs) due to their inner porosity (Supplementary Figure 7d-f).[22,23] However, analogous to metal nanoparticles, it is often observed for porous functional materials that their functionality is concentrated on their outer surface, and not in their inner porosity.[4] As a result, some evidence of similar structure sensitivity behavior for porous functional materials can be found in literature.[24–27]

Traditionally, structure sensitivity studies have been limited to surface science techniques, such as Scanning Tunneling Microscopy (STM) or Low-Energy Electron Diffraction (LEED), as it can be applied to (low index) single crystal samples of metals.[28,29] However, STM operates under strict sample and condition limitations, known as the materials- and pressure gap, respectively. For example, the materials gap restricts measurements to conductive single crystal model systems only, thereby hindering the extrapolation of its results to complex 3D materials, and preventing analysis of porous functional materials with low conductivity altogether.[30,31] Additionally, the pressure gap limits STM operation to vacuum conditions, several orders of magnitude below industrial conditions, thereby often failing to describe guest-host interaction at relevant conditions.[32,33] Bridging of these gaps was achieved for

metal NPs in, for example, the Fischer-Tropsch and Sabatier reaction by performing bulk (IR) studies on metal NPs with a highly defined size distribution at industrial conditions.[18,19,34,35]

However, when applying bulk spectroscopic techniques, such as infrared spectroscopy, dispersion in bulk/surface atoms will result in a loss of information on porous functional material performance. One of the first efforts made to bridge this gap has been performed by Roeffaers *et al*., who showed with *in situ* Single-Molecule Fluorescence (SMF) microscopy that a single edge on an otherwise pristine crystal facet could largely influence the performance of a layered double hydroxide.[36] During the catalytic conversion of individual organic molecules, they found that while transesterification occurred all over the large (0001) terrace, the hydrolysis of the same molecule only occurred on a (1010) edge present within the (0001) terrace. Using conventional bulk techniques this edge plane would have been overlooked, thereby leading to an incorrect interpretation of the performance of the (0001) facet. Whereas this SMF technique is an excellent approach, it does need fluorescent molecules, which limits application for the understanding of small molecules sorption and activation.[37] Therefore, we describe the use of *in situ* PiFM, a surface-sensitive technique that combines the power of infrared spectroscopy for adsorbate studies, with a high spatial resolution able of characterizing high index planes with low-surface coverage on ZIF-8 crystals.

## Formaldehyde partial pressure calculations

Pierce™ 16% formaldehyde (w/v), methanol-free was used as a source of FA by bubbling dry N2 (5.0 purity) through a saturator filled with the FA solution. The FA concentration in the liquid corresponds to 16 mg of formaldehyde (30.026 g/mol) per 100 mL of solution (density 1.04 g/mL at 25 °C), equal to a molar fraction of $9.8 \times 10^{-2}$. According to liquid-vapor equilibrium of water/FA mixtures reported in literature, this equals to 0.45 Torr, or 606 ppm FA in the saturated gas phase at atmospheric pressure and 25 °C (Supplementary Figure 8).[38,39] The concentration of FA in the PiFM cell was finally varied by diluting a FA/$H_2O$ saturated $N_2$ stream with pure $N_2$, to reach a final FA pressure of 60, 120, 180, 240, 300, 360, 420 and 480 ppm.

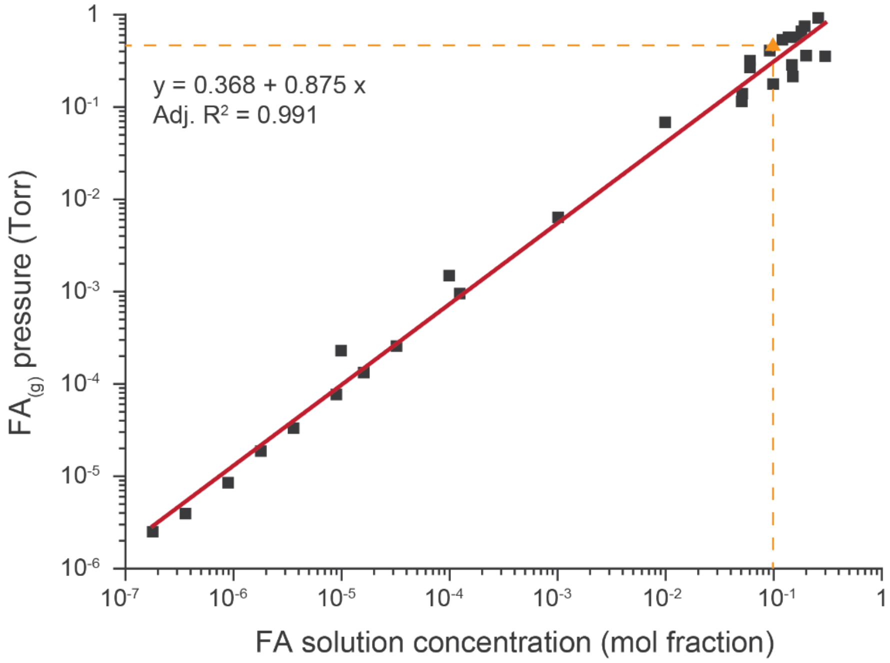


**Supplementary Figure 8.**

Liquid/vapor equilibrium concentration of formaldehyde/water mixtures as a function of liquid concentration. Data are retrieved from [38,39], and the conditions of this study are shown for comparison, as calculated from the linear regression shown in the inset.

**X-Ray Diffraction**

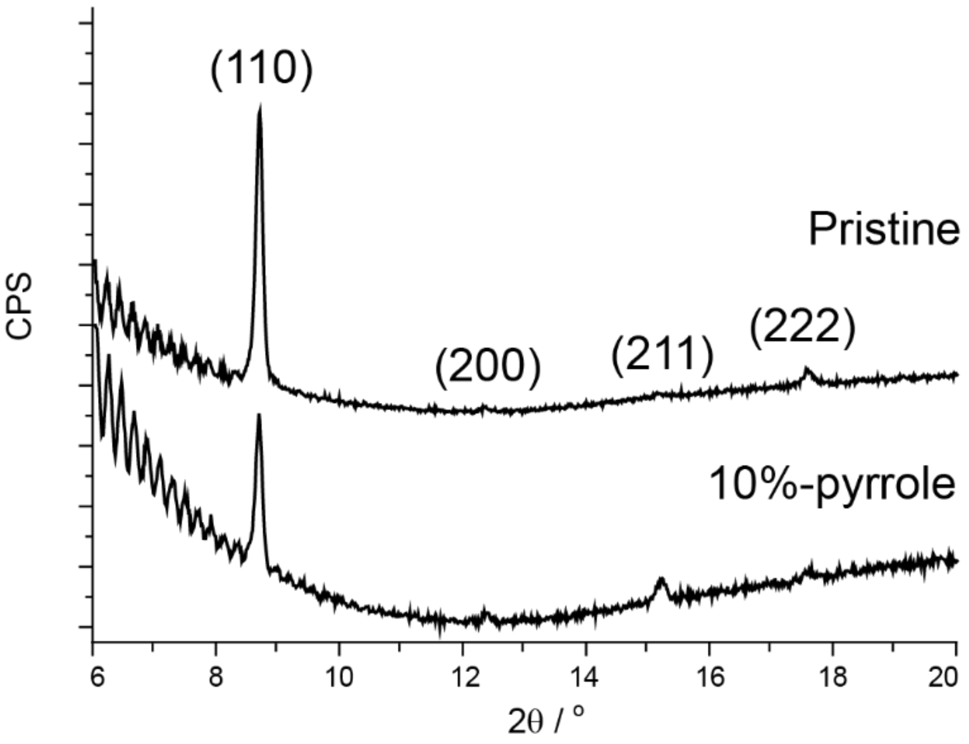


**Supplementary Figure 9.**

XRD patten of pristine ZIF-8 (top) and 10% pyrrole defective ZIF-8 (bottom). The diffractograms confirm the crystalline character of the surface-anchored ZIF-8.

X-Ray Diffraction (XRD) experiments were performed to prove the crystallinity of ZIF-8 deposited through the layer-by-layer synthesis. The diffractograms of the surface-anchored samples show the oriented growth of ZIF-8 in the {110} direction. This oriented growth is the result of using a layer-by-layer synthesis in combination with a gold substrate functionalized with a growth-orienting self-assembling monolayer, namely 4-mercaptopyridine.[40–42] Upon incorporation of 10% pyrrole as defect linker, the diffractogram slightly changes. AFM images (Supplementary Figure 10) showed a change in aspect ratio between crystal planes upon defect-engineering. This change in aspect ratio is reflected in the XRD data.

**Crystal facet aspect ratio of (defect-engineered) SURZIF-8**

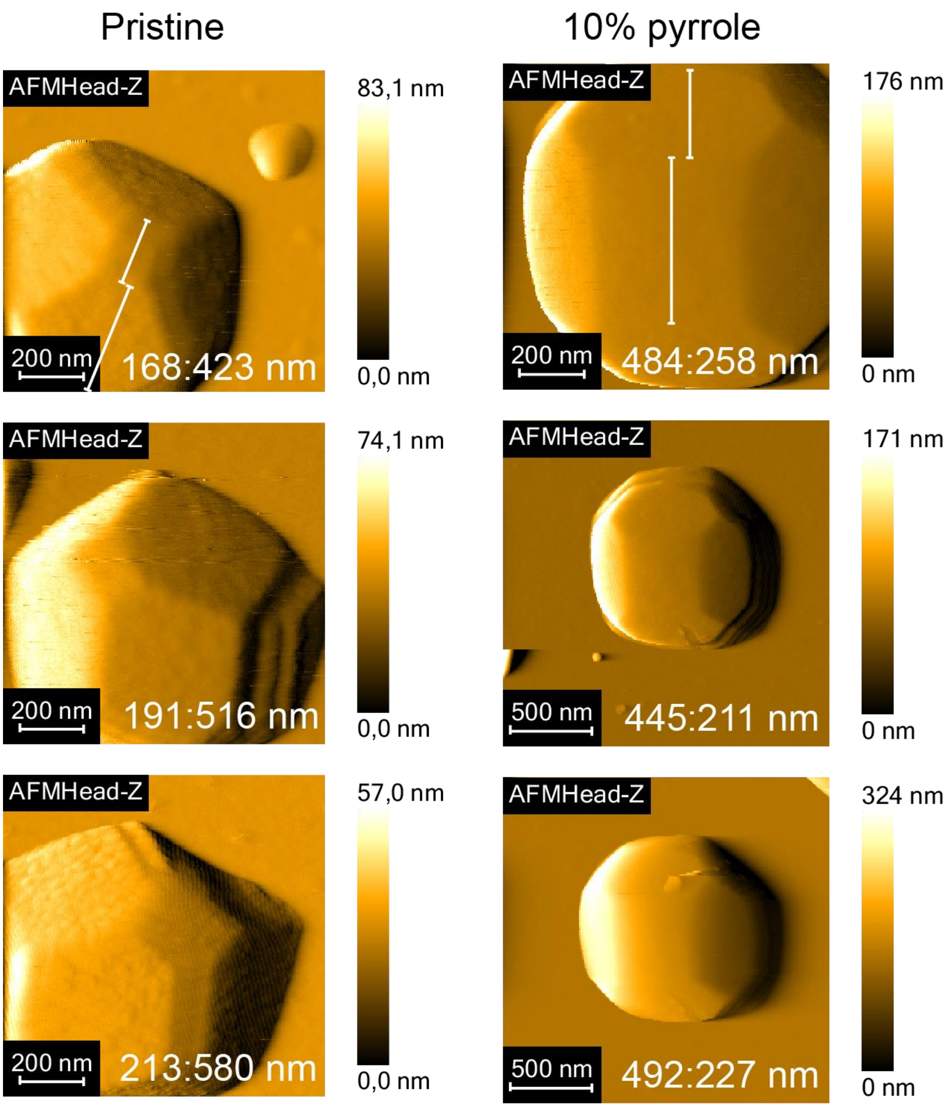


**Supplementary Figure 10.**

AFM images of pristine (left) and defective-ZIF-8 crystals (right). Insets are the ratio between the length of the {100} square edge and the length of the adjacent {110} facet parallel to the used square edge. Upon incorporation of 10% pyrrole linker, the size ratio increases.

AFM images of pristine and defective ZIF-8 crystals show the increase in {100}:{110} aspect ratio. DFT calculations showed the preferential incorporation of defective linkers into high-energy crystal planes. In pristine ZIF-8, the {100} plane is higher in energy than the {110} plane, resulting in a low aspect ratio. Upon defect incorporation, the surface energies of both facets are lowered, however, the higher-energy {100} plane is affected more than the {110} plane, resulting in the observed change in crystal aspect ratio.

**Masking of hyperspectral images**

Using PiFM, we were not limited by the diffraction spot of IR, since the tip acts as an antenna for IR light, amplifying the signal from a nm-size spot on the sample. We were thus able to obtain an IR spectrum for every pixel of the microscopy images reported in the paper, together with the corresponding topographic image of the same crystal. This was crucial, since it allowed us to describe both the surface-averaged spectrum of the ZIF-8 crystal surface, by averaging the IR spectra of all these pixels, as well as allow us to separate spectral contributions of different crystal planes. To do so, we used the topography map, which was acquired simultaneously with the IR image, to create masks corresponding to a crystal plane, and finally averaged spectra in such regions.
We divided the acquired hyperspectral images into relevant areas (facets, edges, corners) to compare inter-plane sorption and conversion characteristics. To ensure comparability, it was chosen to acquire hyperspectral images of the full ZIF-8 crystals (rather than perform zoom-in measurements of specific areas) and mask the relevant locations. An example of such a hyperspectral image, and the corresponding phase image to show sample morphology, is given in Supplementary Figure 11.

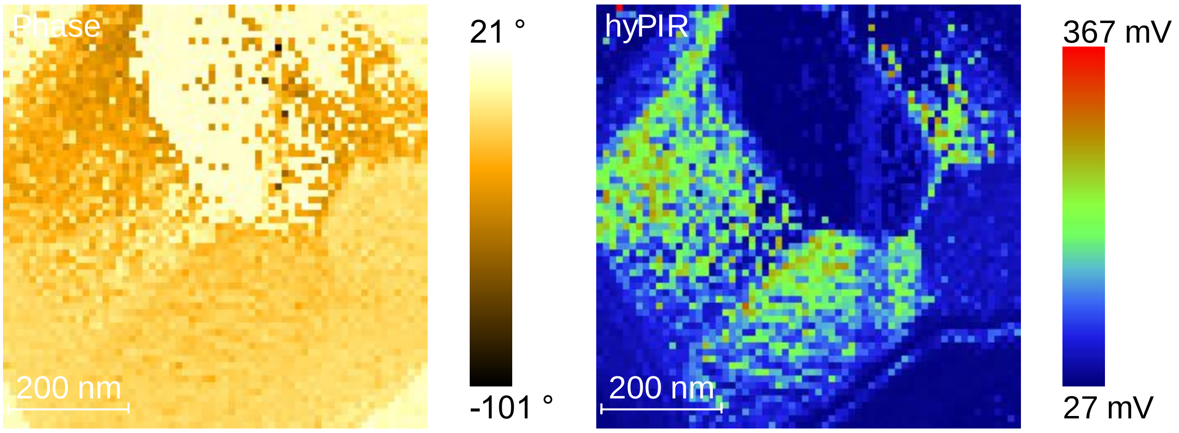


**Supplementary Figure 11.**
Phase image (left) and hyperspectral image (right) of a pristine ZIF-8 crystal at 120 ppm of formaldehyde pressure. Phase images were used as visual guide to segment the hyperspectral images for inter-plane comparison of formaldehyde sorption and conversion behavior.

The different crystal planes can readily be recognized in the phase image, as well as the hyperspectral image. This phase image was used as a guide to draw masks for the planes,

edges and corners, as well as remove IR signal from the gold substrate. The following subsections show the masks that were drawn for the facets, edges, and corners for this specific hyperspectral image. This methodology was applied to hyperspectral images of the pristine and defective crystals at each formaldehyde pressure.

Facet masks

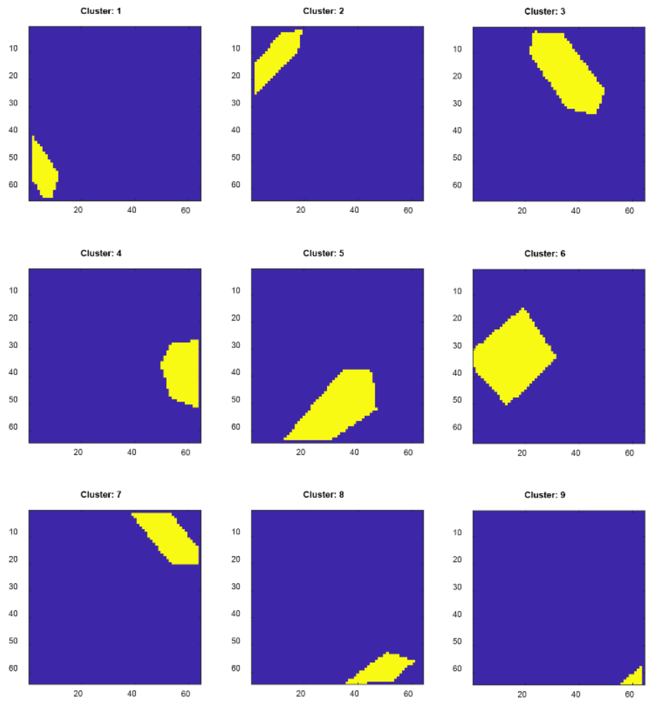


**Supplementary Figure 12.**

Masks applied to the hyperspectral image shown in Supplementary Figure 11b to filter out spectral information describing the {110} facets (mask #1-5) and the {100} facets (#6-8). Mask #9 filtered out an area of the gold substrate on which the ZIF-8 crystal was grown.

Supplementary Figure 12 shows the masks apply to filter out the {100} and {110} facets. To improve the S/N ratio, the average spectrum of a masked area was used to describe the spectral identity of the crystal plane. Masks resulting in spectral outliers were discarded. The remaining masked spectra were averaged to describe the averaged behavior of the {100} and the {110} plane during formaldehyde sorption. This methodology was also applied to the masks used to describe the edge (Supplementary Figure 13) and corner (Supplementary Figure 14) planes.

<u>Edge masks</u>

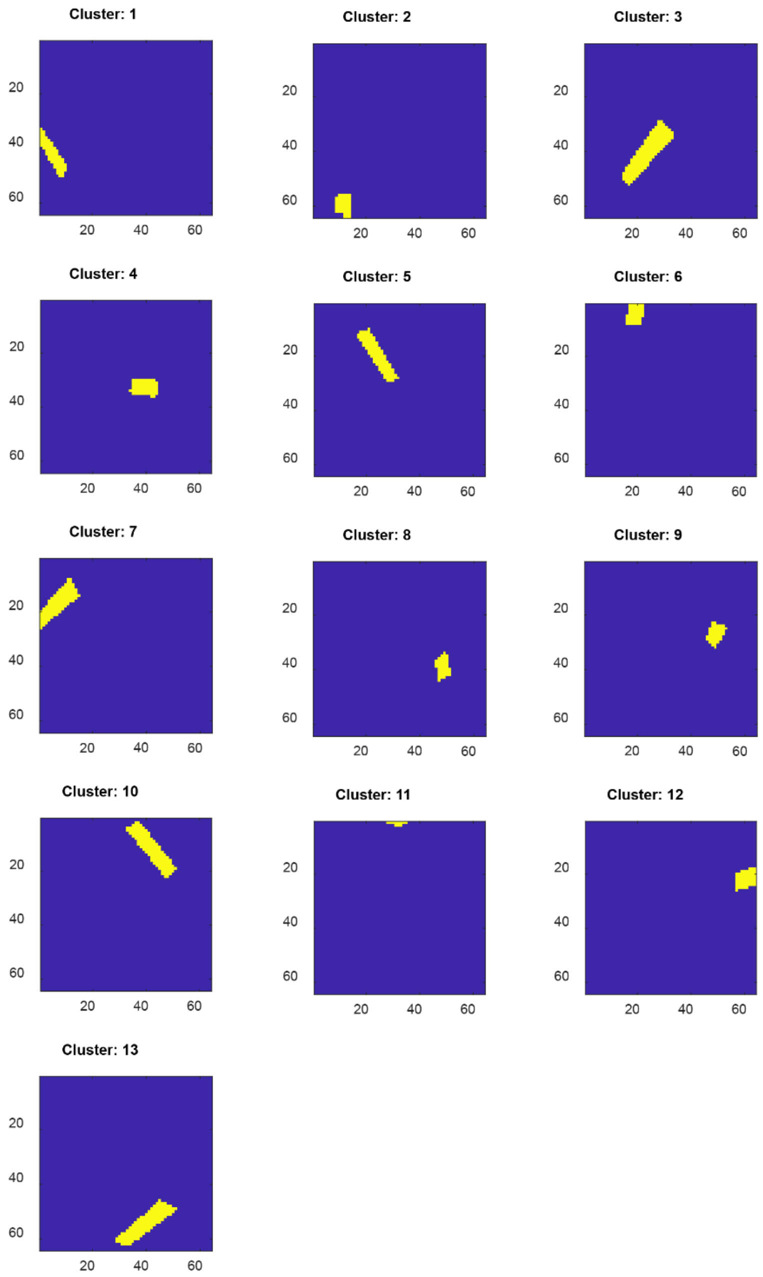


**Supplementary Figure 13.**

Masks applied to the hyperspectral image shown in Supplementary Figure 11b to filter out spectral information describing the {210} edges (mask #1, 3, 5, 7, 10, and 13) and the {211} edges (#2, 4, 6, 8, 9, 11, and 12).

Corner masks

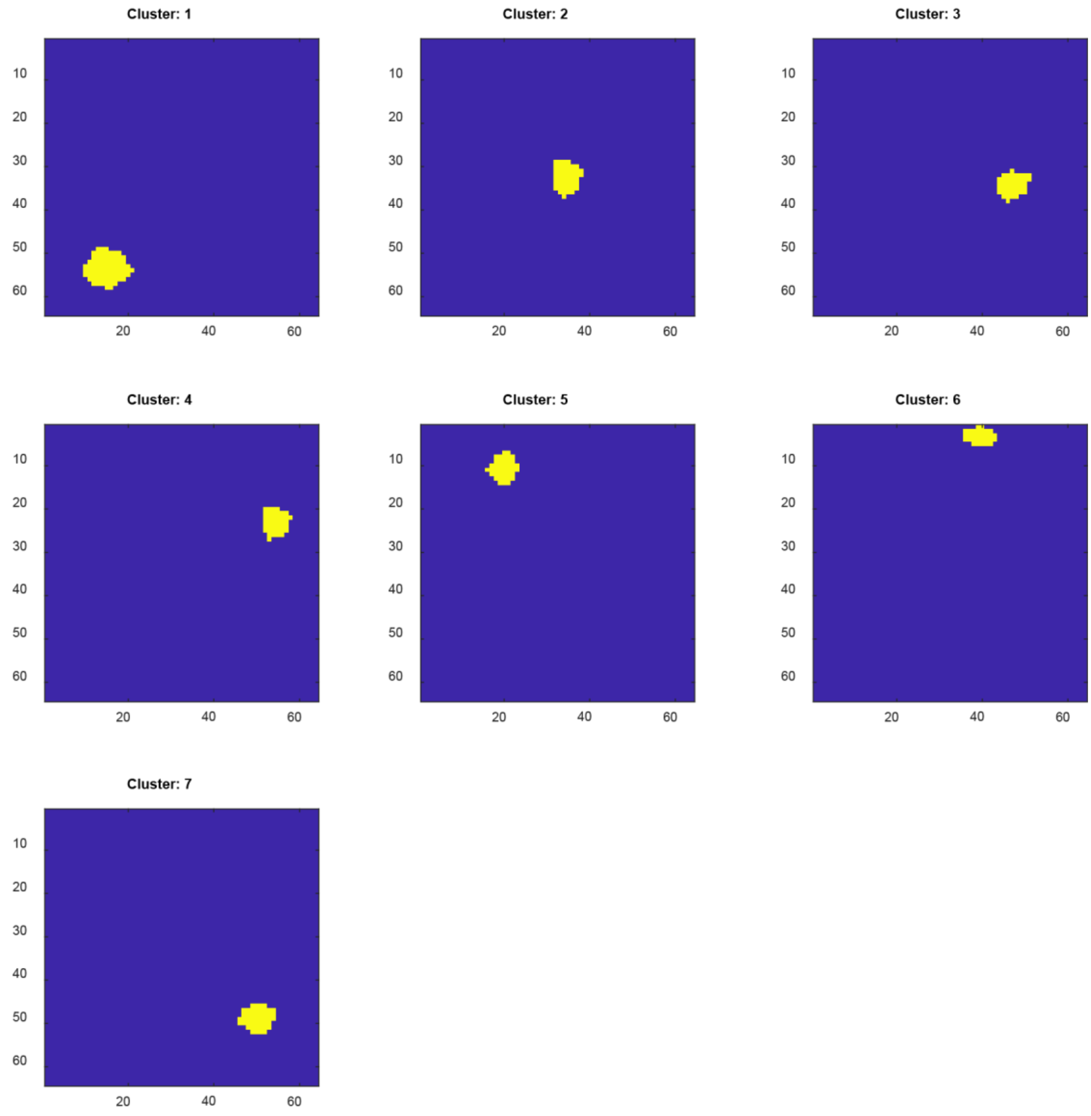


**Supplementary Figure 14.**

Masks applied to the hyperspectral image shown in Supplementary Figure 11b to filter out spectral information describing the {111} corner (mask #3) and the {310} corners (#1, 2, 4-7).

## Hyperspectral images of pristine ZIF-8 during in situ PiFM measurements

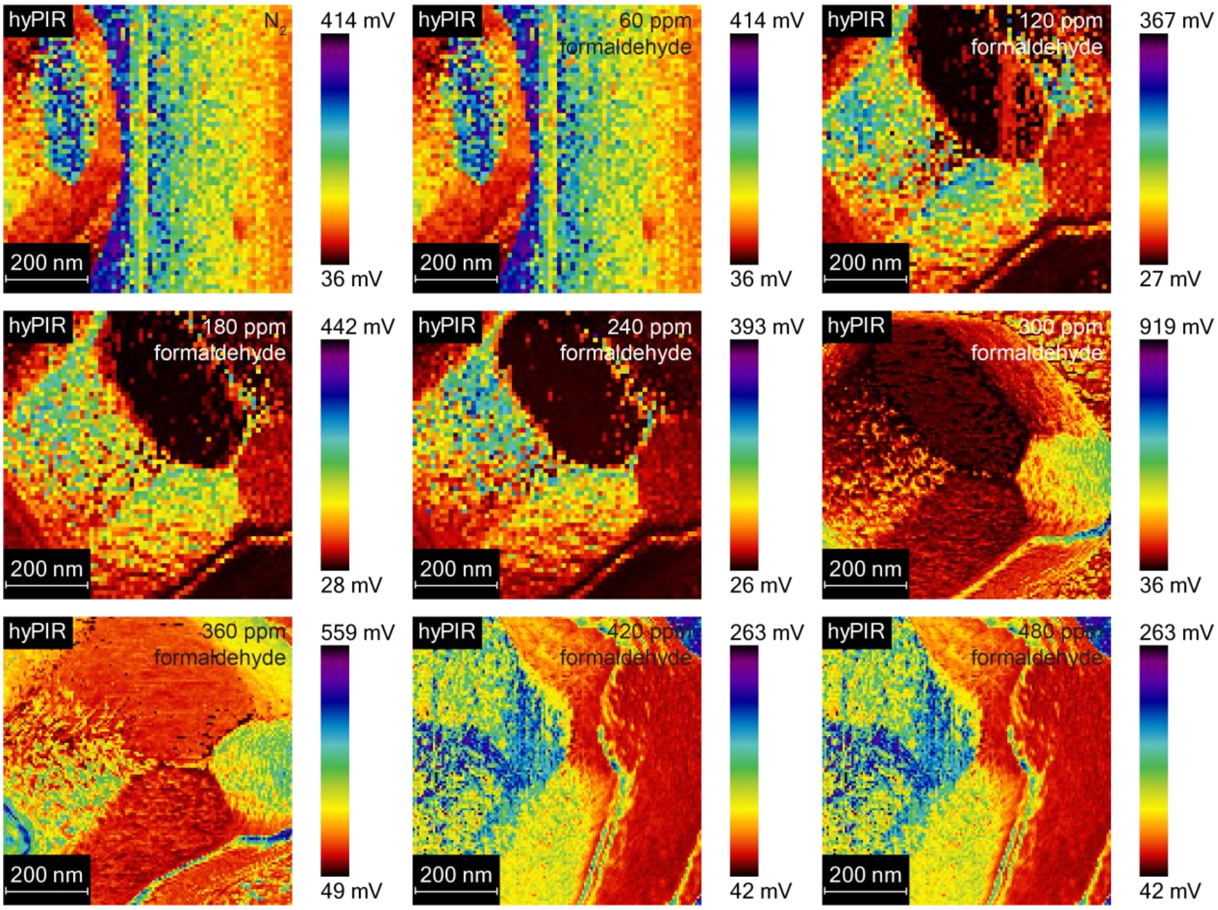


**Supplementary Figure 15.**

Hyperspectral images of pristine ZIF-8 crystals at increasing formaldehyde pressures, where each pixel represents a full (1965-775 $cm^{-1}$) IR spectrum. Scalebars reflect the IR signal intensity of the most intense IR band within each spectrum. An image resolution of 64 x 64 pixels was chosen to ensure fast data collection and to minimize drift effects (examples of drift are found in the two top left images leading to compression of the ZIF-8 crystal in the image).

## *In situ* PiFM on pristine ZIF-8

Contour plots in situ formaldehyde sorption on pristine ZIF-8

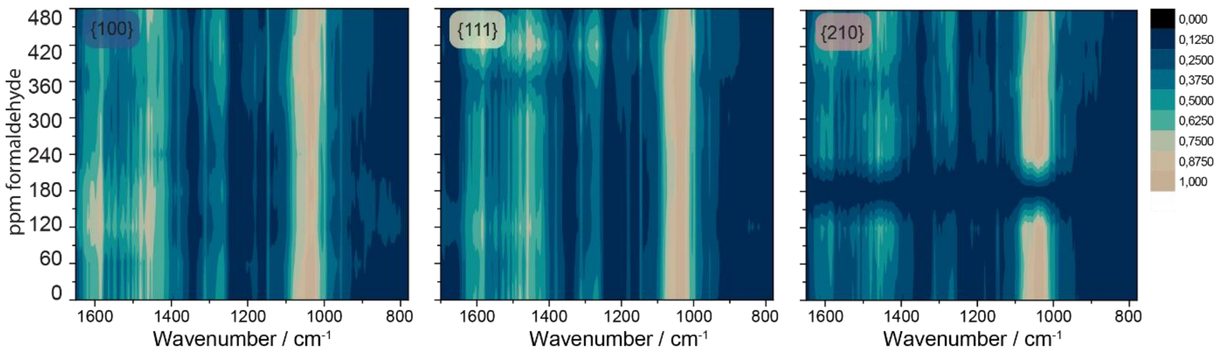


**Supplementary Figure 16.**

Contour plots showing the *in situ* formaldehyde sorption behavior of the {100}, {111}, and {210} planes of a pristine ZIF-8 crystal. These plots complement the contour plots shown in Figure 2F-H. For the {210} plane, no data at 120 ppm of formaldehyde pressure was available leading to a gap in the contour plot. As a guide, the contour plots interpolate between datapoints ($\Delta p$ = 60 ppm) to witness the transition in IR spectrum upon formaldehyde adsorption.

Masked *in situ* hyperspectral spectra

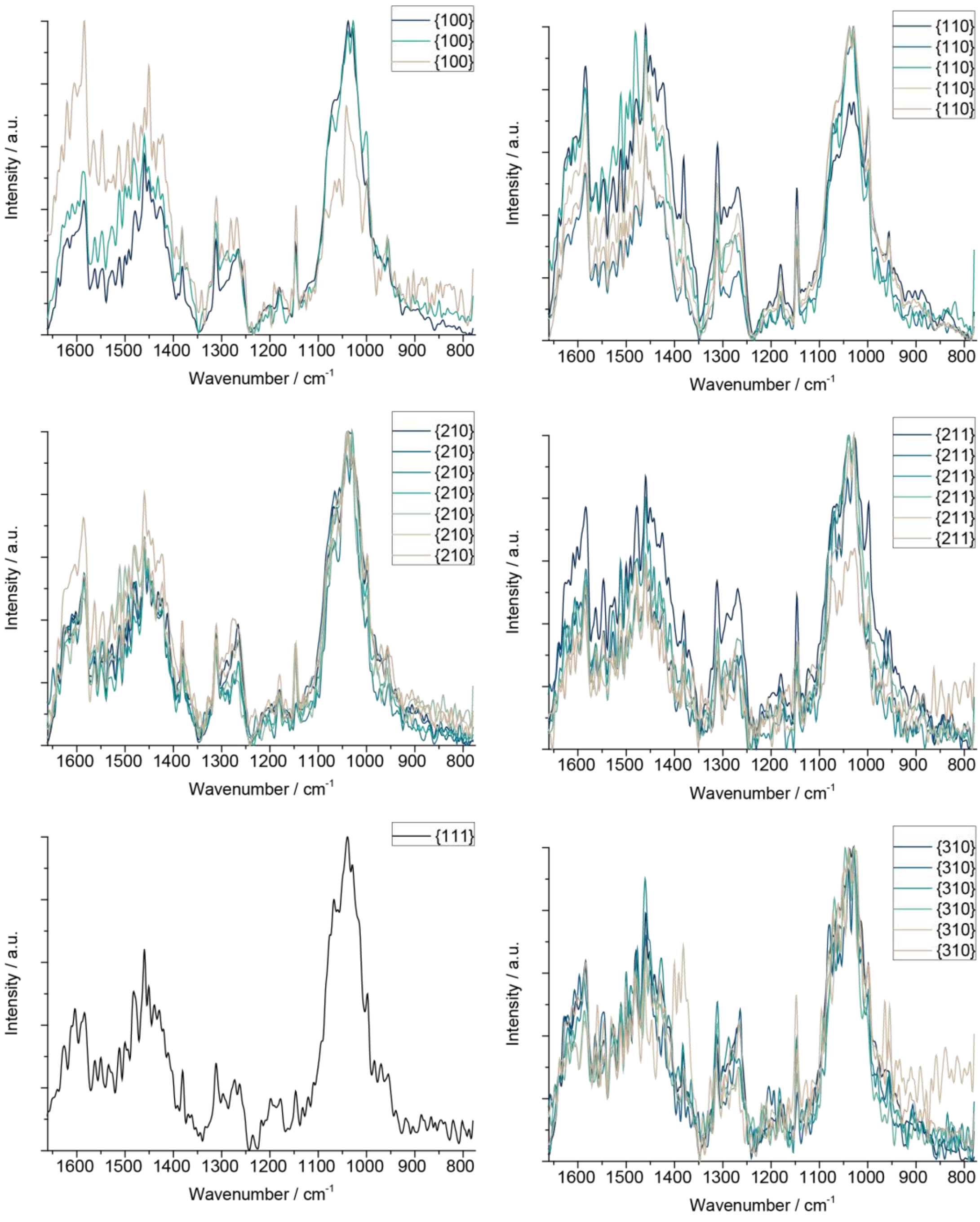


**Supplementary Figure 17.**

Mask-averaged spectra corresponding to the masks shown in Supplementary Figures 12-14. The hyperspectral image was recorded for a pristine ZIF-8 crystal at 180 ppm of formaldehyde pressure.

Pressure-dependent crystal plane averaged IR spectrum

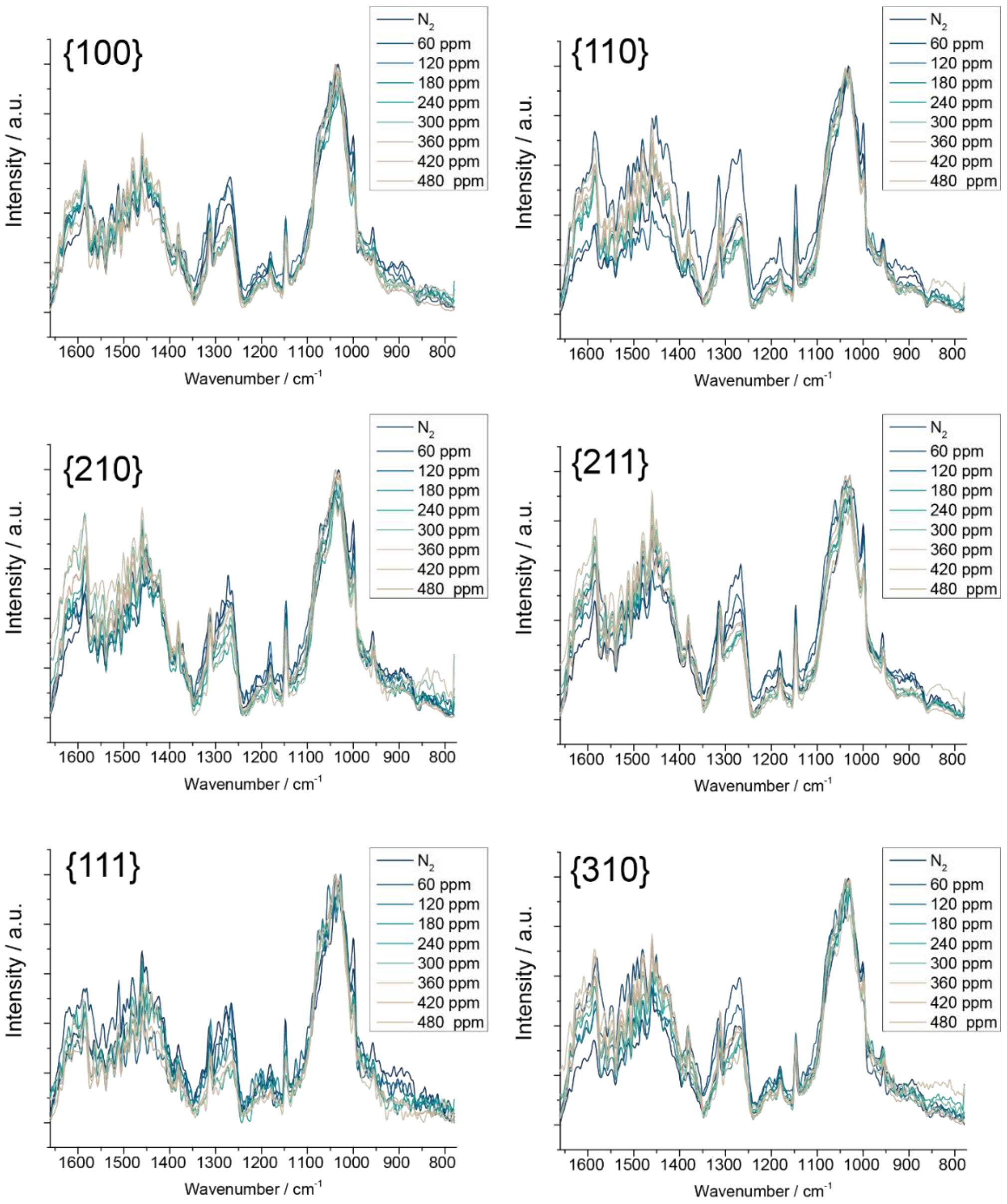


**Supplementary Figure 18.**

Crystal plane-averaged IR spectra as a function of increasing formaldehyde pressure for a pristine ZIF-8 crystal. Crystal plane-averaged spectra were constructed by averaging all mask-averaged spectra of each crystal plane. This was done to improve the S/N ratio and to facilitate inter-plane spectrum comparison. This information was used to construct Figure 2F-I.

In situ plane averaged difference spectra

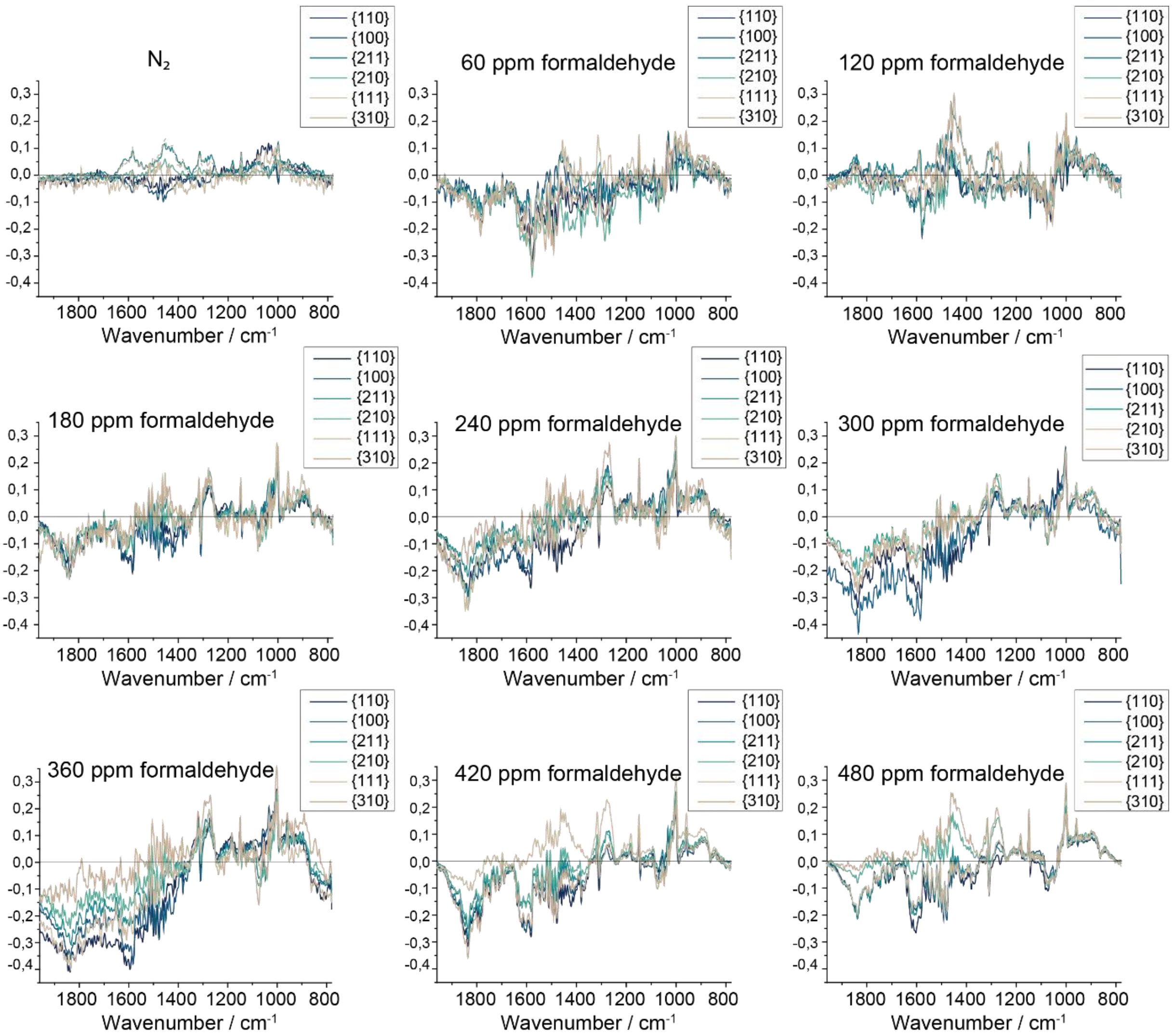


**Supplementary Figure 19.**

Pressure-dependent difference spectra for the crystal planes of a pristine ZIF-8 crystal, where the spectra of crystals in nitrogen were subtracted from the spectra of crystal planes in formaldehyde. The difference spectra highlight the rise of formaldehyde bands at increasing VOC pressure and the faster response of high-index planes, such as the {310} corner planes. This information was used to construct Figure 2I.

**Table of infrared band assignments**

**Supplementary Table 4.**

A table listing the assignment of infrared bands of (defective) ZIF-8 and formaldehyde(-derived) species.

| *Wavenumber* | *Assignment* | *Species* |
|---|---|---|
| *~1750* | $\nu_{C=O}$ | Formaldehyde |
| *1280* | $\delta_{CH2}$ rock | Formaldehyde* |
| *1200* | $\delta_{CH2}$ wag | Formaldehyde* |
| *896* | $\nu_{C-O}$ | Formaldehyde* |
| | | |
| *1580* | $\nu_{COO-}$ asym. | Formate* |
| *1380* | $\nu_{COO-}$ sym. | Formate* |
| *1320* | $\tau_{CH2}$ | DOM/POM* |
| *1150* | $\nu_{C-O}$ | DOM/POM* |
| *1060* | $\nu_{C-O}$ | Methoxy* |
| | | |
| *1590* | $\nu_{C=N}$ | ZIF-8 |
| *1461* | $\nu_{C=C}$ arom. | ZIF-8 |
| *1310* | $\delta_{C=C}$ arom. | ZIF-8 |
| *1148* | $\nu_{C-N}$ | ZIF-8 |
| *1100* | $\nu_{C-N}$ | ZIF-8 |
| *995* | $\delta_{NH}$ | ZIF-8 |
| | | |
| *1160* | $\delta_{N-D}$ | ZIF-8 Pyrrole-$d_5$ |
| *960* | $\delta_{C-D}$ | ZIF-8 Pyrrole-$d_5$ |
| *885* | $\nu_{C=N}$ | ZIF-8 Pyrrole-$d_5$ |
| | | |
| *840* | $\delta_{OH}$ | Zn-OH |
| *790* | $\delta_{OH}$ | Zn-OH |

## Mapping of intra-facet heterogeneity

Additional point spectra within PiFM maps

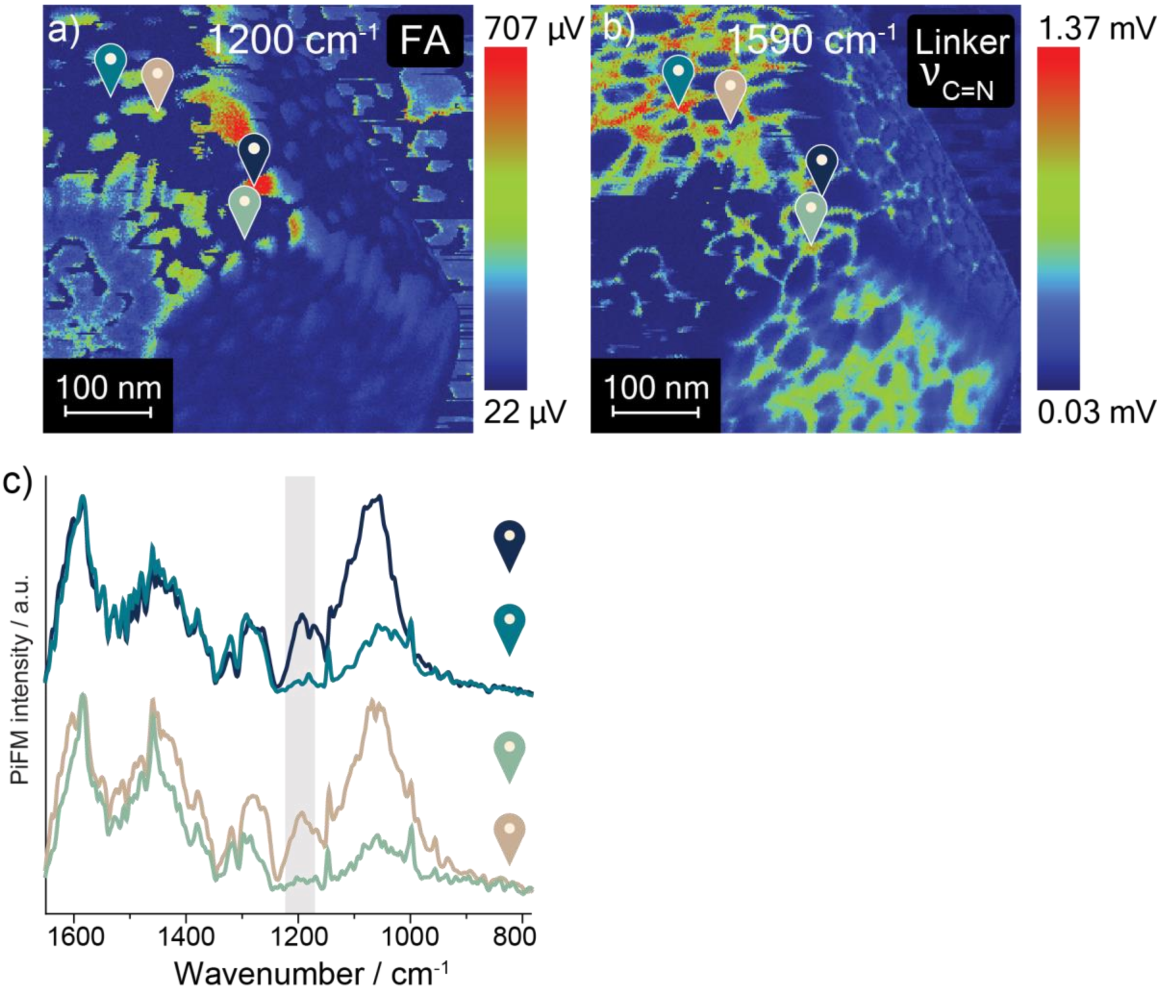


**Supplementary Figure 20.**

Additional point spectra taken at 300 ppm FA pressure within the IR maps shown in a, b) and Figure 4A, B. These point spectra show stable ZIF-8 spectra with variable FA band intensities dependent on location within the IR maps. These spectra show the power of the mapping procedure in *in situ* PiFM to visualize heterogeneity in functional materials in gas vapor.

*In situ* infrared mapping of ZIF-8 crystals in formaldehyde vapor

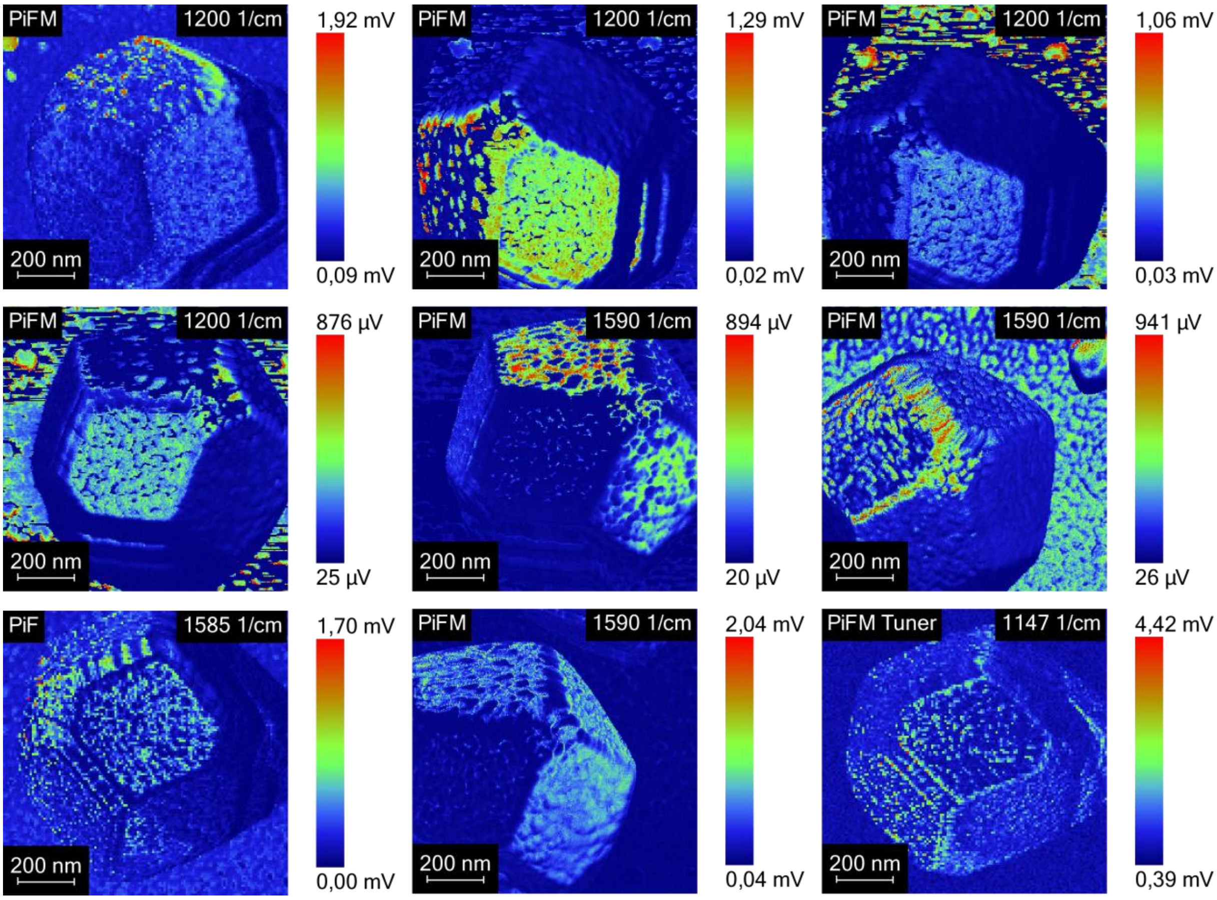


**Supplementary Figure 21.**

Additional IR maps of several ZIF-8 crystals in 300 ppm of formaldehyde vapor. The wavenumber used during the measurement is listed in the inset of each image. These images underline the applicability of the IR mapping function in *in situ* PiFM to detect non-homogeneous sorption behavior on the nanoscale.

**Full PiFM spectra PCA and clustering analysis**

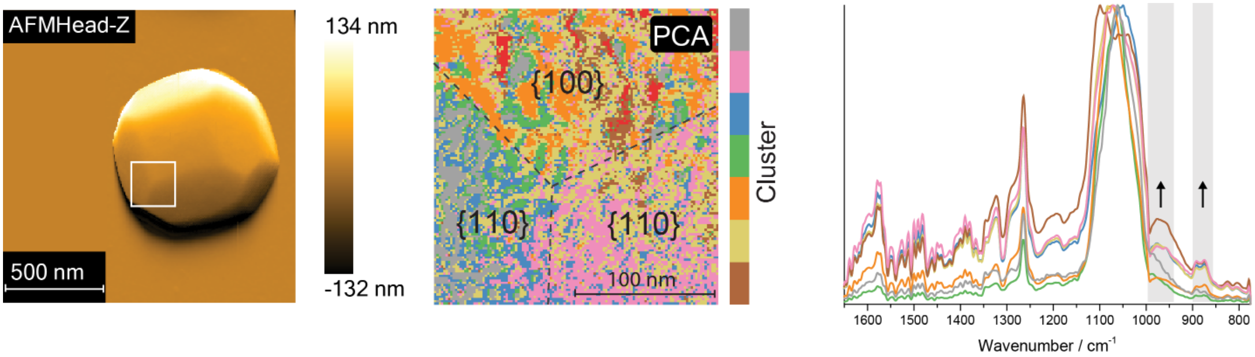


**Supplementary Figure 22.**

Full spectra normalized to the highest band intensity corresponding to the PCA spectra shown in Figure 4G. Bands marked by the grey boxes belong to vibrations of the defective pyrrole linker. Peak ratios between various ZIF and pyrrole bands were calculated for each of the spectra.

**Size analysis intra-facet nano-islands**

Size analysis was performed in Fiji using the NanoDefine plugin “Particle Sizer” (PS) analyzer.[43] The PS script was developed to automatically measure the distributions of the characteristic size and shape properties of a nanomaterial. Compared to usual particle analysis in ImageJ, where a threshold is applied to the global image, the plugin uses a local threshold, estimated for a specific circular region with the configured radius. This allows for a better discrimination of even partially overlapping domains.

The domain distribution for the defect-engineered ZIF-8 crystal was evaluated from the clustered image reported in Figure 4F, which was converted to grey scale and analyzed using the default parameters of the PS plugin (Supplementary Figure 23). Since only part of the image was analyzed (Supplementary Figure 23B), the analysis was repeated on the inverted image (Edit/Invert; Supplementary Figure 23C). Supplementary Figure 23D reports the overlap of the two analyzed images, showing the entire image was analyzed, with only a few islands being analyzed twice. The resulting size distribution is consistent between images B and C (Supplementary Figure 23E, F) and results in an overall broad distribution, with an average of 63 $nm^2$ size and a large standard deviation of 61 $nm^2$. This is the case for all the size analyses, due to the large deviation observed in the size of the domains. Nonetheless, we can qualitatively compare the samples looking at the shape of the size distribution and comparing their average domain size.

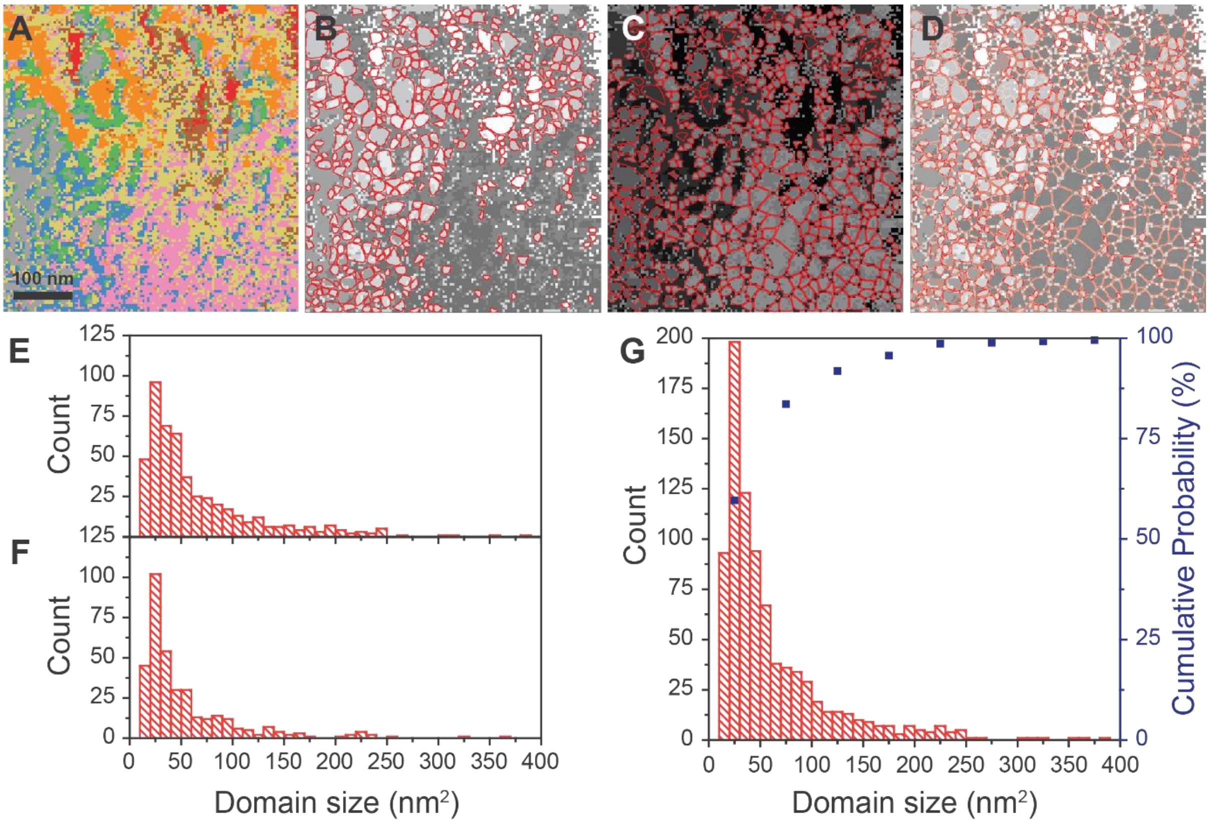


**Supplementary Figure 23.**

A) clustered image used for size analysis. B) derived grey scale image, with analysis results from the Particle Sizer (PS) plugin shown as outlines. C) inverted image analyzed by PS. D) overlay of panels B and C, showing complete analysis of the image. E,F) domain size analysis for images B and C respectively, showing comparable distributions. G) sum of size distribution of domains obtained from the two analysis.

The size analysis was repeated using the irregular watershed convexity threshold in the PS plugin (Supplementary Figure 24). This threshold determines if a particle is counted as primary or not based on the particle convexity. If the convexity is lower than a threshold (set to 0.7 as default), the particle is split, otherwise it is not. The average size of the domains obtained with this method is 129 nm$^2$, due merging of some of the smaller domains observed with default settings. Since the outlines of the identified domains better match the clusters of the original image, this analysis was considered more successful, and used for comparison with the pristine ZIF-8 crystal case.

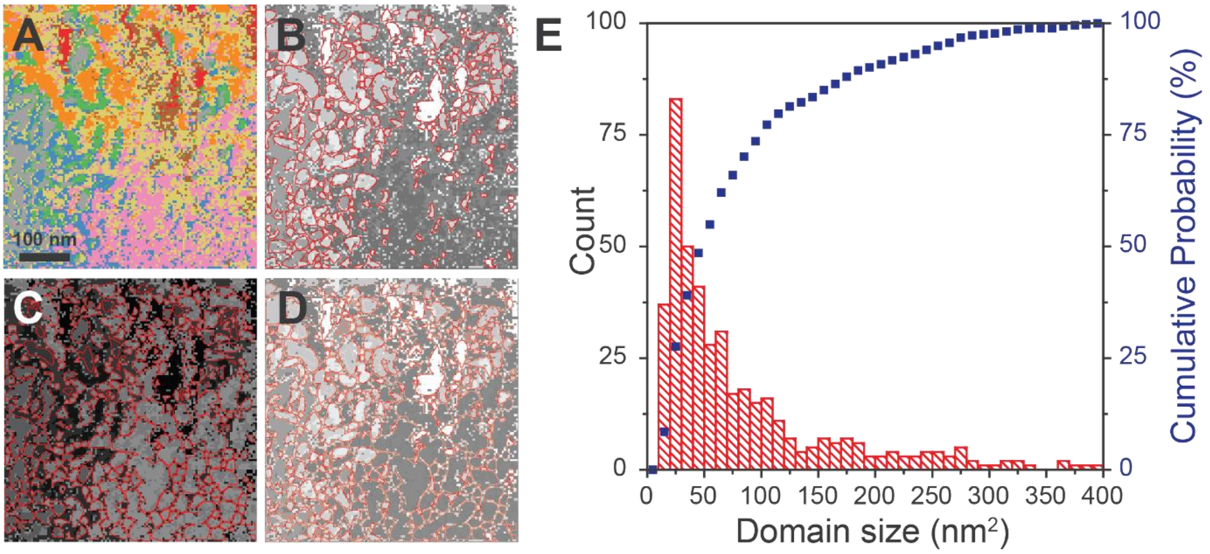


**Supplementary Figure 24.**

A) clustered image used for size analysis. B) derived grey scale image, with analysis results from the Particle Sizer (PS) plugin using the default irregular water threshold (IWT) option shown as outlines. C) inverted image analyzed by PS-IWT. D) overlay of panels B and C, showing complete analysis of the image. E) domain size analysis results from images B and C, combined.

The same analysis procedure was applied to the *in situ* PiFM IR maps of pristine ZIF-8 reported in Figure 4A, B. The size distribution is similar in the two cases, with an average domain size of 153 and 189 $nm^2$ for the 1590 $cm^{-1}$ (Supplementary Figure 25A) and the 1200 $cm^{-1}$ (Supplementary Figure 25B).

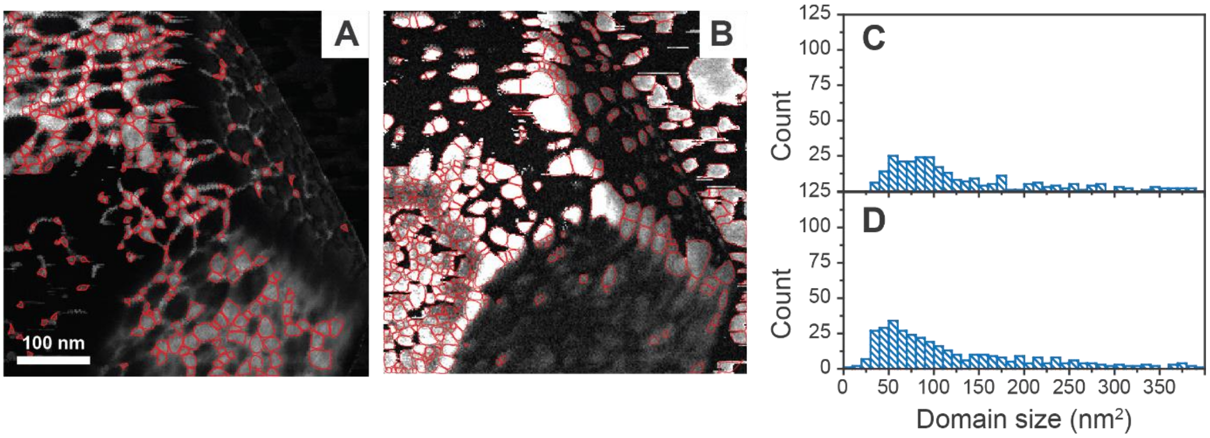


**Supplementary Figure 25.**

A) 1590 $cm^{-1}$ IR map (aromatic vibrations from the ZIF-8 framework) with analysis results from the Particle Sizer (PS) plugin using the default irregular water threshold (IWT) option shown as outlines. B) 1200 $cm^{-1}$ IR map (formaldehyde signals) analyzed by PS-IWT. C,D)

overlay of panels B and C, showing complete analysis of the image. E) domain size analysis for images A and B respectively, showing comparable distributions (average size: 153 and 189 $nm^2$).

We finally compared the analysis results obtained for the pristine and defective crystals, as shown in Supplementary Figure 26. The main difference in the domain size distribution lies in the higher percentage of smaller islands being detected in the case of the defective crystals. Looking at the cumulative probability of the domain size, we can identify a similar trend, with 75 % of domains having an area below 150 $nm^2$, and 99% below 400 $nm^2$, in both cases.

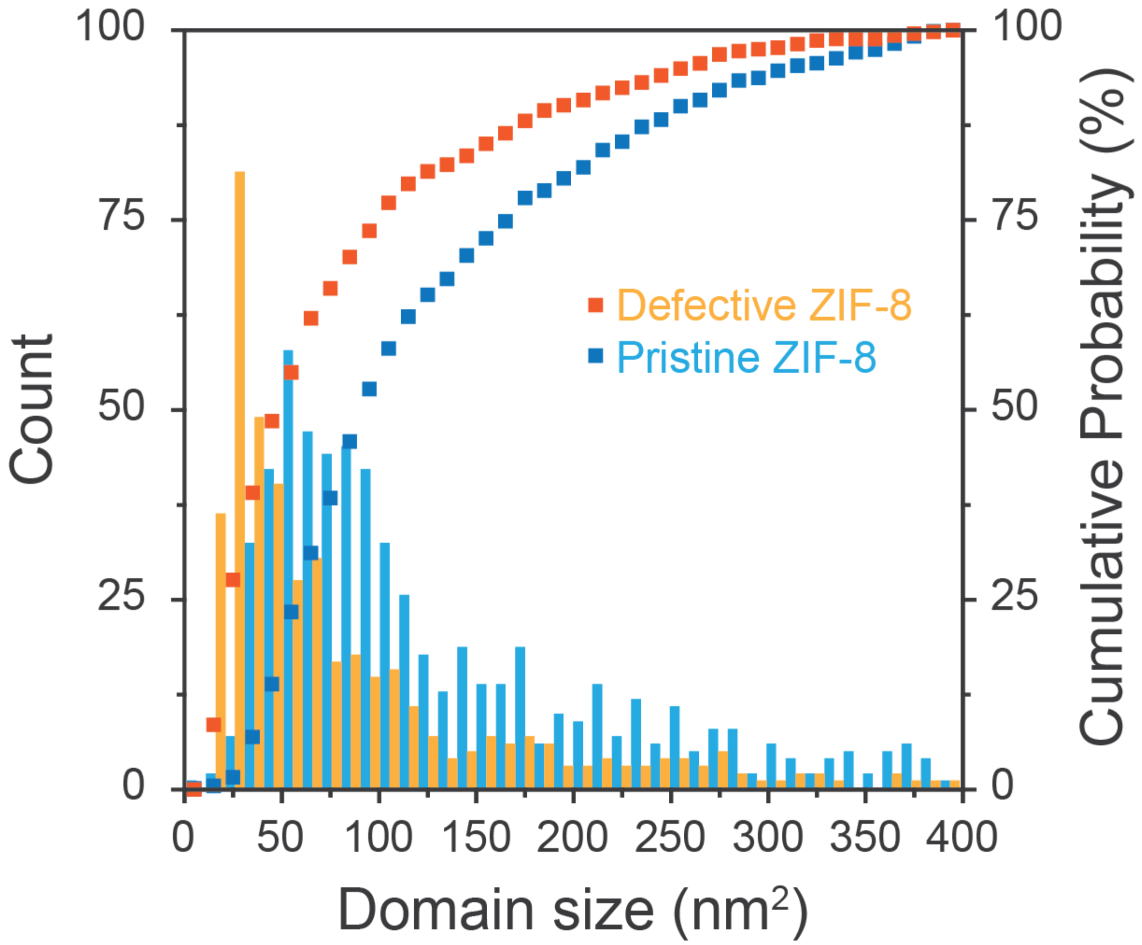


**Supplementary Figure 26.**

Domain size analysis of pristine and defective ZIF-8 crystals, showing similar size distributions and cumulative probability (average domain area: 176 and 129 $nm^2$, respectively).

### *In situ* PiFM on defective ZIF-8

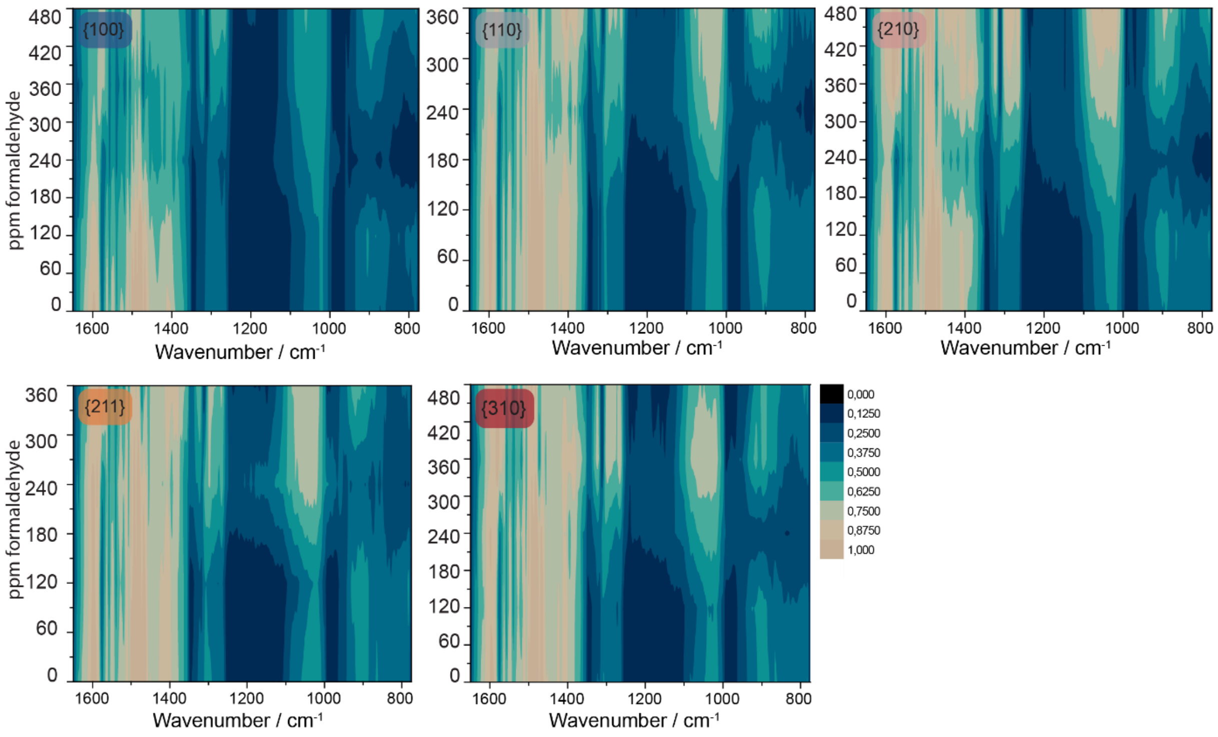


**Supplementary Figure 27.**

Contour plots of *in situ* PiFM measurements on defective ZIF-8 crystals. Plots are divided to show crystal plane specific information. No information was available for the {111} plane due to a change in crystal plane aspect ratios upon defect incorporation. Vibrations corresponding to adsorbed and converted formaldehyde were found for all crystal planes. However, the pressure at which these IR bands appeared differed between crystal planes.

**Formaldehyde adsorption and conversion response pressures on defective ZIF-8 areas with high-/low-defect concentrations**

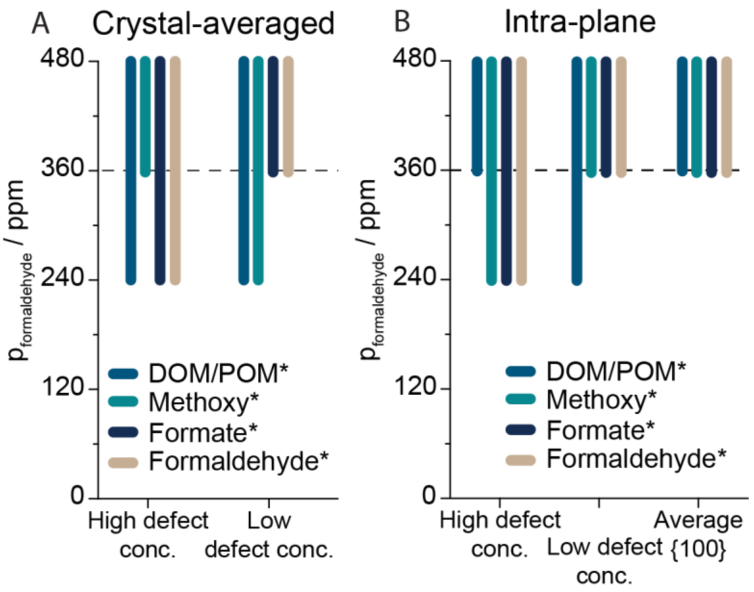


**Supplementary Figure 28.**

(**A**) Crystal-averaged response pressures for formaldehyde-related species over defect-rich and defect-poor surface fractions (bulk response shown with dashed line) show that a higher defect concentration did not necessarily equal favored FA sorption/conversion. However, within a single {100} plane the defective nanodomains were shown to exhibit a faster response to FA (conversion) than the pristine sites (**B**). This underlines the necessity for nano-scale *in situ* analysis of functional materials to correctly establish structure-performance relationships.

To disentangle the contributions of crystal plane terminations and defect-induced Lewis acidity we used nano-spectroscopy to distinguish their effects on the structure sensitive conversion of formaldehyde. To do so we constructed the plots in Supplementary Figure 29A-C where we compared the response pressures, i.e. the pressure at which IR bands of DOM/POM, formate, and methoxy species appeared in the IR spectrum, on the crystal surface-averaged, inter-plane, and even intra-plane scale. We constructed these plots by averaging the IR spectra of relevant hyperspectral image pixels per FA pressure, and subsequently inspecting these spectra for signs of surface species formation. This information was translated to the plots, where the onset of the vertical plot lines signal the FA pressure at which the surface species were formed.

## Intra-facet Principal Component Analysis and clustering of the {100} plane of defective ZIF-8

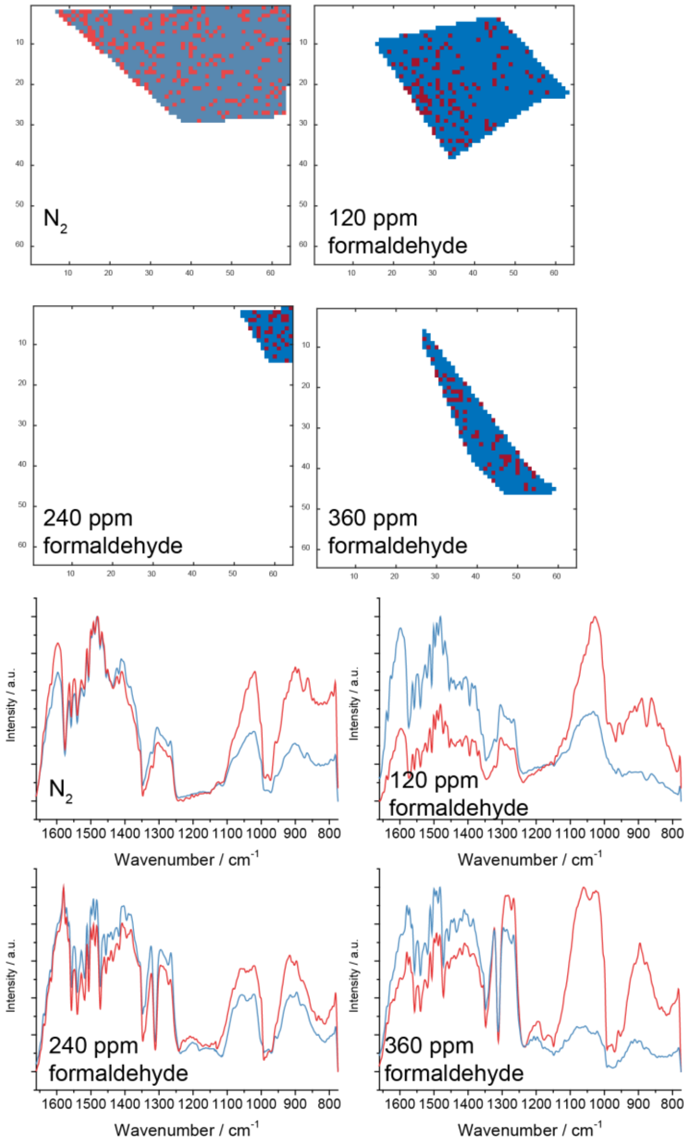


**Supplementary Figure 29.**

Segmentation of some {100} planes over increasing formaldehyde pressure. The planes were segmented into defect-rich (red) and defect-poor (blue) areas. The spectra corresponding to these segments are plotted below. Using these spectra, the intra-plane influence of defect sites on formaldehyde sorption and conversion could be found.

## Principal Component Analysis and clustering of hyperspectral images of defective ZIF-8

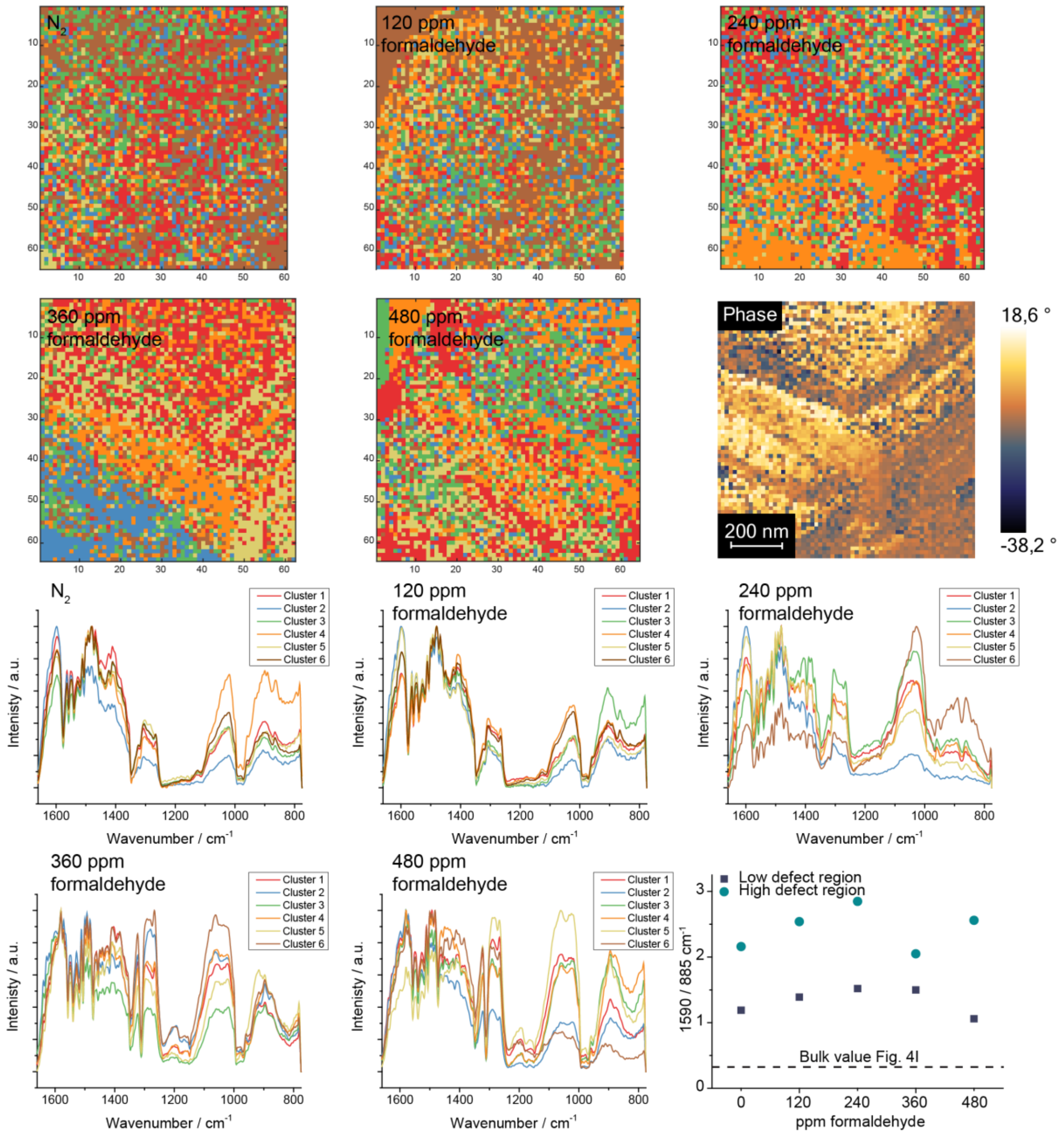


**Supplementary Figure 30.**

Examples of clustered hyperspectral images (created through Principal Component Analysis and clustering) of defective ZIF-8 crystals at increasing formaldehyde pressure. The spectra belonging to the clusters are shown below. PCA and clustering was applied to hyperspectral image to identify defect-rich and defect-poor regions. This approach was used to find defect regions spanning the whole crystals, rather than being confined to specific crystal planes. To find these regions we calculated ZIF/pyrrole peak ratios for all clusters. The highest and lowest ratio per pressure is plotted together with the bulk value found for the defective crystal

in Figure 4E, I. Higher overall values were found since a crystal with high coverage with high-index planes was selected to increase spectrum quality for these planes. This information was used to construct Figure 4C.

**DFT calculations on formaldehyde sorption on defective ZIF-8 surfaces**

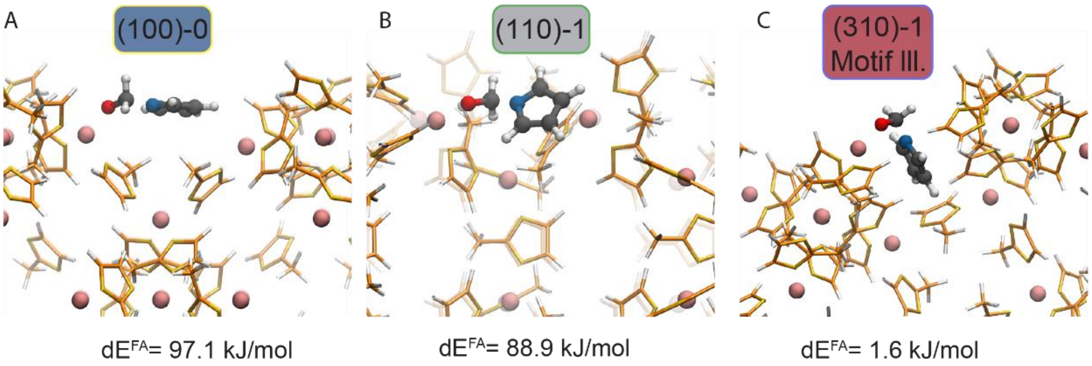


**Supplementary Figure 31.**

(**A-C**) DFT calculation results showing defect-induced creation of Lewis acidity on all surface terminations. This Lewis acidity resulted in the creation of new ZIF-8 surface ensembles for FA sorption, leading to alternate binding conformations, lowered binding energies and ultimately in formaldehyde conversion. Furthermore, DFT calculations showed that while the introduction of defects resulted in lowered binding energies of formaldehyde on all planes, the adsorption (and conversion) of FA remained structure sensitive in nature.

**FA adsorption on ZIF-8 followed by *in situ* Attenuated total reflectance (ATR) FTIR spectroscopy**

To study FA adsorption on ZIF-8 crystals at the bulk scale, ZIF-8 powders and thin films were deposited on ATR crystals and exposed to FA *in situ*. ATR configuration was chosen because transmission FTIR spectroscopy of ZIF-8 pellets showed strong interferences with the water and FA vapor, yielding low quality data.

<u>Preparation of ZIF-8 samples on Si ATR crystals</u>

ZIF-8 samples were prepared by LbL synthesis according to the procedure described in the first section of the SI (see MOF deposition), with the only difference that Si ATR crystals were used instead of Au substrates.

To allow for more sample to be deposited on the ATR crystals and enhance the signals from adsorbed species, ZIF-8 powders were prepared using a solvothermal method in methanol at room temperature: pristine ZIF-8 powders were obtained by dissolving 405 mg $Zn(NO_3)•6H_2O$ (98%, Sigma-Aldrich) and 263 mg 2-methylimidazole (99%, Sigma-Aldrich) each in 20 mL methanol (99.9%, Sigma-Aldrich). Both solutions were combined and stirred at 1000 rpm for 5 min and afterwards left without stirring over night at room temperature. The white powders were collected via centrifugation and washed three times with methanol and subsequently dried overnight at 50 °C. For the synthesis of defective ZIF-8 crystals (dZIF-8), 10 mol% of 2-methlyimidazole was replaced by pyrrole (98 %, Sigma Aldrich). Besides the replacement of the linker-containing solution by a solution of 237 mg 2-methylimidazole and 22 mg pyrrole, the subsequent synthetic steps remained the same as for the pristine ZIF-8. Additionally, a commercial ZIF-8 sample (ACS materials, 500 nm crystal size) was used as a reference.

To prepare the samples for ATR experiments, the synthesized or commercial ZIF-8 powder was suspended in methanol, drop-casted on a Si ATR crystal and dried overnight at 50 °C.

All materials were characterized by SEM, revealing the formation of sub-micrometre crystals in all cases (Supplementary Figure 32). The pristine and defective ZIF-8 powders were further characterized by FTIR spectroscopy and XRD, which confirmed the formation of the MOF crystal structure (Supplementary Figure 33).

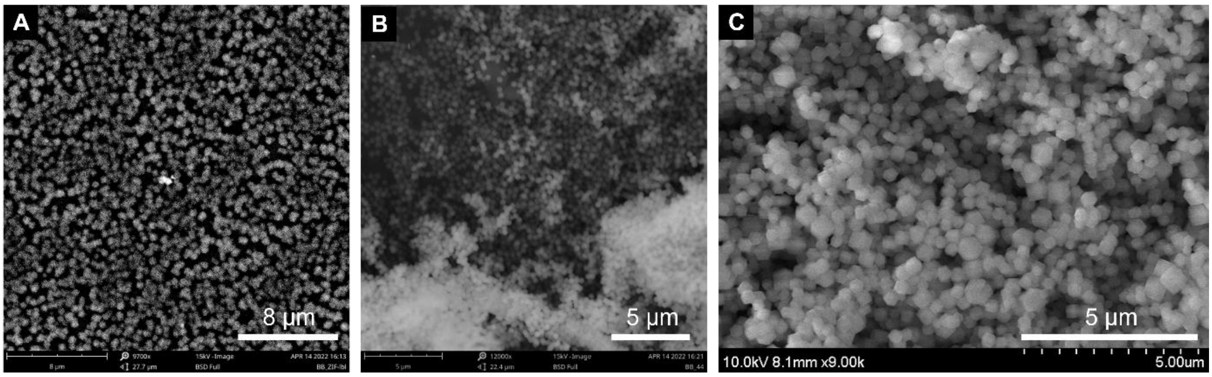


**Supplementary Figure 32**. SEM images of ZIF-8 crystals synthesized (A) by LbL on a Si ATR crystal, and (B) by the solvothermal method. (C) SEM image of the commercial ZIF-8 crystals.

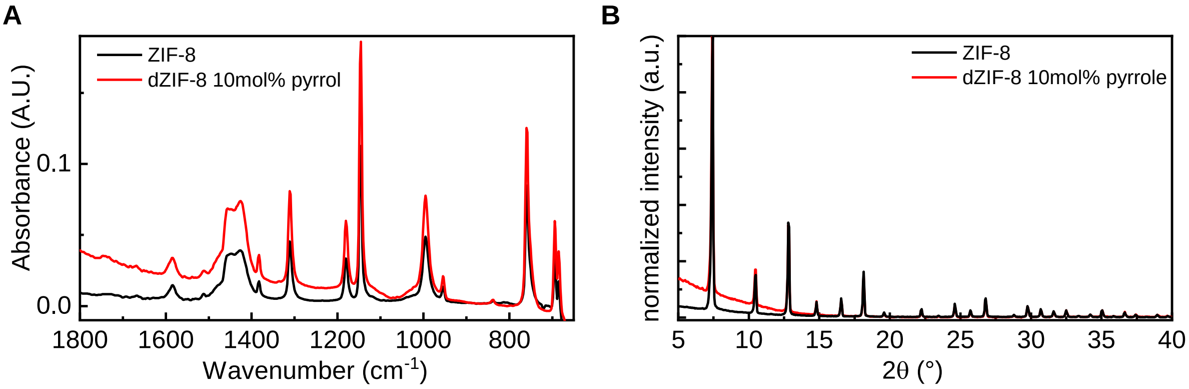


**Supplementary Figure 33**. ZIF-8 powder characterization via FTIR spectroscopy (A) and XRD (B).

<u>*In situ* FTIR-ATR cell for FA and methanol adsorption studies</u>

A custom-made gas flow cell and ATR setup, adapted from refs.[44,45], was placed in a Perkin Elmer Three FTIR spectrometer equipped with a $N_2$-cooled MCT detector (Supplementary Figure 34). 1/8 inch PP tubing was connected to the gas flow cell using M5 adapters (Festo). For each spectrum 128 scans were averaged. ATR crystals (20 x 10 x 0.5 mm, 45°) cut from double side polished Si wafer and a depth of penetration $d_p$ = 0.52 µm ($\lambda$=1500 $cm^{-1}$, n(Si) = 3.42, n(ZIF-8)=1.3) and an effective pathlength of $d_{e\parallel}$=0.66 µm $d_{e\perp}$=0.33 µm, yielding at total effective pathlength of $\frac{d_{e\parallel}+d_{e\perp}}{2}\cdot N$= 9.89 µm with N=20 were used.

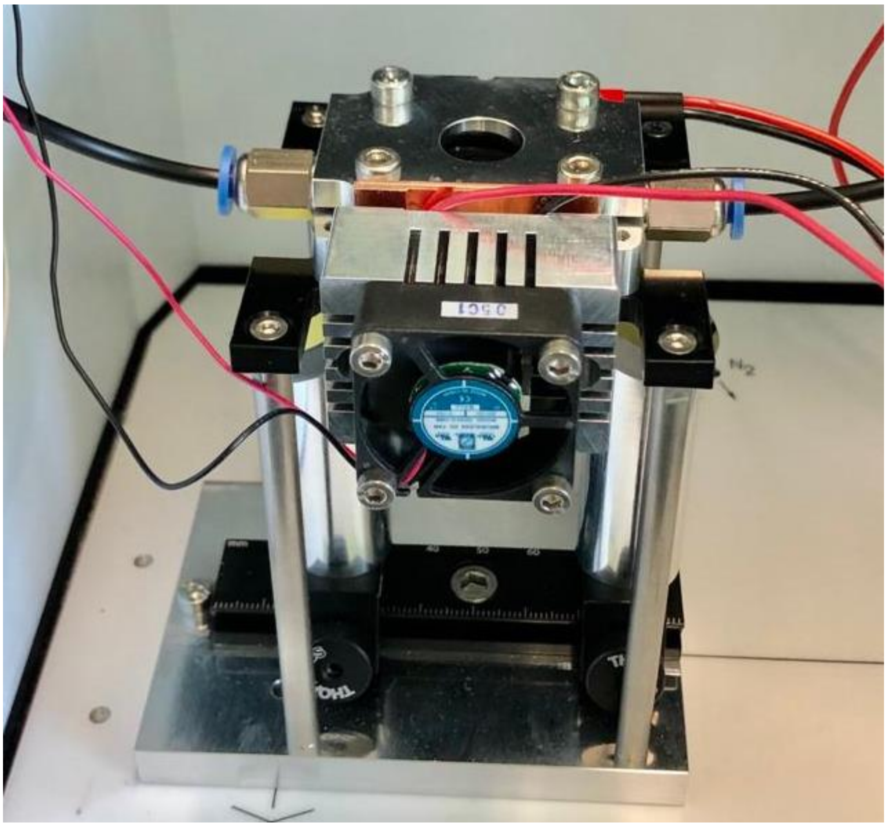

**Supplementary Figure 34.** Photograph of the ATR flow cell used in this study.

FA or methanol vapours were introduced in the cell at various partial pressures by mixing a dry $N_2$ flow with a saturated $N_2$ flow, obtained by bubbling $N_2$ through liquid FA or methanol at room temperature. The flows were controlled by means of mass flow controllers (Bronkhorst), to achieve a total flow of 100 mL/min. The actual concentrations were determined by transmission IR spectroscopy in a 10 cm transmission cell with ZnSe windows. For calibration of FA and methanol vapours, transmission spectra were integrated in the C-H stretching region between 3100–2800 $cm^{-1}$. Concentrations were obtained from the band areas using reference spectra of 1 ppm/m FA or methanol from the PNNL database.
Before each FA adsorption experiment, the cell was flushed with pure $N_2$ for 10 min. Subsequently, the FA partial pressure was increased by 0.02 steps in a range of $p/p_0$ = 0 – 0.1, and in intervals of 0.1 for higher pressures, and kept for 3 min at each step to reach equilibrium.

<u>Methanol adsorption isotherm measured via *in situ* FTIR-ATR reveals gate opening of ZIF-8</u>
ZIF-8 is known to undergo structural changes upon adsorption of vapours of alcohols, water and acetone, so-called "gate opening".[46,47] Such deformations result in an increase in the ZIF-8 pore volume after exposure to certain critical concentrations of guest molecule vapour,

and were associated with S-shaped isotherms during adsorption, not only for ZIF-8, but also ZIF-4, ZIF-7, and ZIF-9.[47] For ZIF-8, the critical vapour concentration at which an abrupt increase in absorption and penetration of the porous volume was observed due to gate-opening was reported as 10, 5, and 3 vol.% for methanol, ethanol and propanol respectively.[46,47] The critical pressure was further observed to decrease with increasing molecular weight of the alcohol, with butanol having a critical pressure of around 0.1 %.[47] Notably, all these concentrations are higher than the maximum FA pressure used in this study (0.05 vol.%). These observations strongly suggest that the signals observed during FA adsorption on ZIF-8, both with PiFM and FTIR-ATR, only stem from species adsorbed on the surface of the crystals, as the pressure is too low to induce gate opening. We therefore here assume that no gate opening takes place during our study.

Prior to FA adsorption, we wanted to gather evidence that the signals are exclusively obtained from the surface of the ZIF-8 crystals and no pore condensation and penetration of the FA takes place. Due to the low achievable FA concentration and the high partial vapor pressures needed to observe the characteristic jump in sorption isotherms upon pore condensation, methanol, as most similar molecule in terms of size and polarity, was used to achieve a broad range of vapor concentrations and to determine the pore condensation step. Supplementary Figure 35 shows the band height of adsorbed methanol as a function of applied methanol concentration on the ZIF-8 materials produced by solvothermal methods and on a commercial ZIF-8 sample. A steep increase in concentration is only observed for > 12000 ppm, which is in line with reported gate opening concentration for ZIF-8[46] and significantly higher than the applied FA concentrations. Notably, all samples showed comparable methanol isotherms, despite their different morphology and size (see SEM in Supplementary Figure 32). Since the commercial sample has a comparable size to the microcrystals synthesized by LbL synthesis (1 µm) used in the PiFM study, we believe the observations can be used to justify our assumption of absence of gate-opening in the FA adsorption study.[48] Therefore, we can assume that the bulk experiments performed using FTIR-ATR spectroscopy at FA concentration < 600 ppm only show features of species adsorbed to the crystal surface, as it is observed with PiFM.

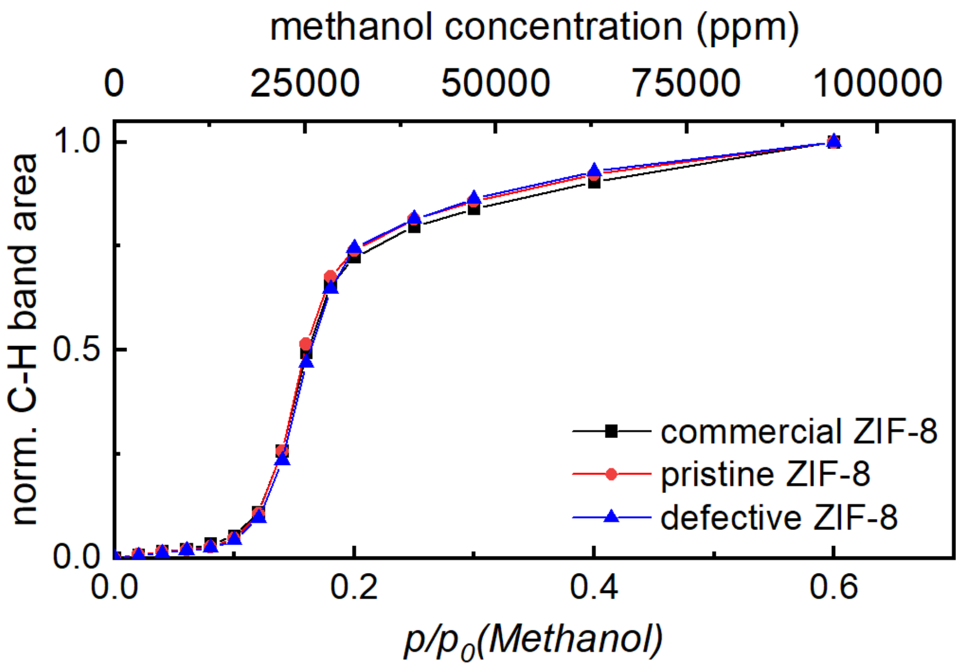


**Supplementary Figure 35**. Methanol isotherm obtained from ATR spectra recorded on the pristine (red dots) and defective (blue triangles) ZIF-8 synthesized by solvothermal method and a commercial ZIF-8 (black squares) for increasing methanol concentrations, showing the typical S-shaped isotherm due to gate opening of ZIF-8.

*In situ* FTIR-ATR FA adsorption experiments

The spectra obtained from the adsorption of FA between 0 – 550 ppm onto commercially available ZIF-8 are shown in Supplementary Figure 36. The negative bands at 1574 $cm^{-1}$, 1480 $cm^{-1}$, and 1303 $cm^{-1}$ arised from band shifts of the ZIF-8 vibrations due to the increasing refractive index of the sample during FA and water adsorption and the associated anomalous dispersion of ATR bands.[45] Starting at a FA concentration of 16 ppm, bands associated with FA and DOM appeared at 1180 $cm^{-1}$ and 1320 $cm^{-1}$. From 63 ppm FA onwards, bands assigned to formate species at 1550 $cm^{-1}$ and 1400 $cm^{-1}$ occurred and increased in intensity for higher FA concentrations, while the intensity for the bands associated with FA and DOM stabilized. These results are consistent with PiFM experiments on defective ZIF-8, where adsorbed FA and DOM were initially observed, followed by formate species (see Figure 5 in the main text).

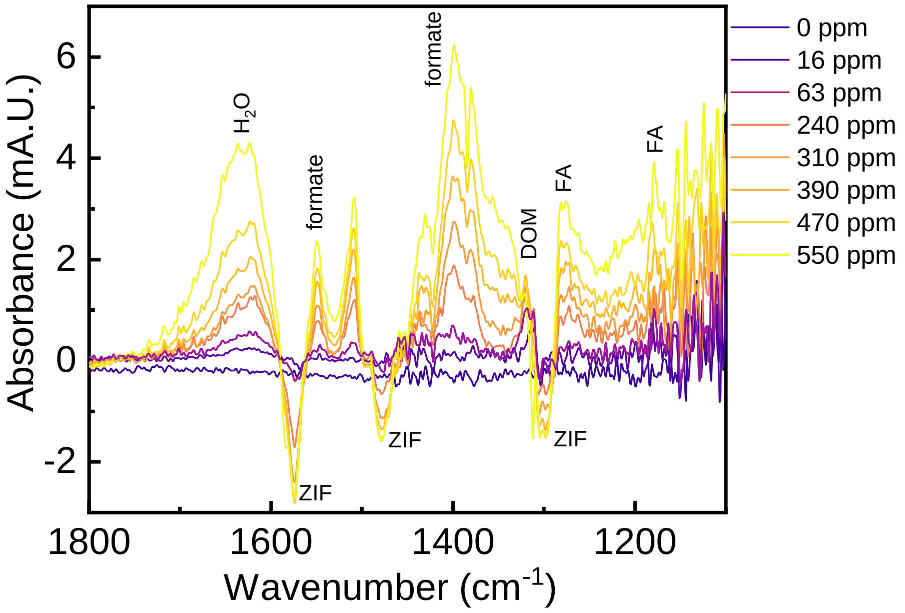


**Supplementary Figure 36**. FTIR-ATR spectra of FA adsorbed on commercial ZIF-8. Background spectrum = $N_2$ flushed ZIF-8 film.

FA adsorption experiments with ZIF-8 prepared in house in methanol at room temperature with and without pyrrole yielded comparable spectral features and trends (Supplementary Figures 37-S38).

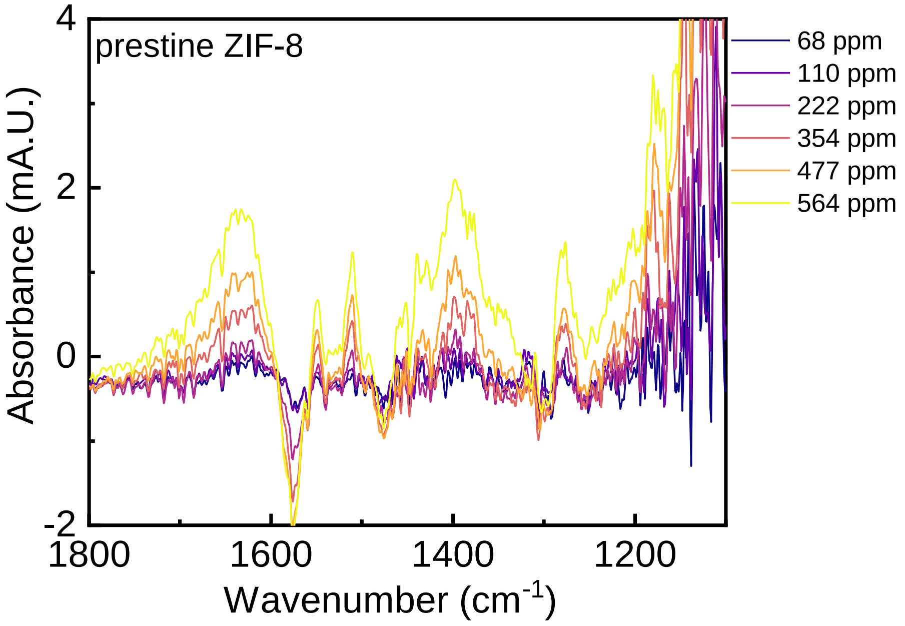


**Supplementary Figure 37**. FTIR-ATR spectra of FA adsorbed on pristine ZIF-8 prepared in methanol. Background spectrum = $N_2$ flushed ZIF-8 film.

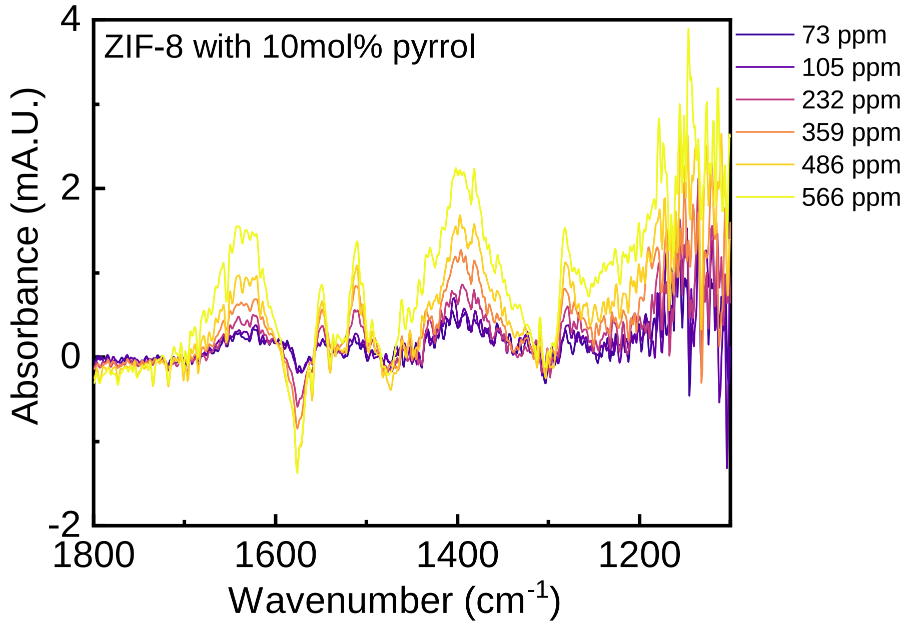


**Supplementary Figure 38**. FTIR-ATR spectra of FA adsorbed on defective ZIF-8 prepared in methanol with 10 mol% pyrrole. Background spectrum = $N_2$ flushed ZIF-8 film.

Due to the low crystal loading of the ZIF-8 prepared on the Si ATR crystal via LbL, the volume probed by the evanescent wave was not covered with ZIF-8 as in the previous cases, but showed strong water and FA vapor lines, which inhibited the analysis in the presence of these gases. Nonetheless, after replacing the FA/$N_2$ atmosphere with $N_2$, comparable bands were found as in the previous cases (Supplementary Figure 39).

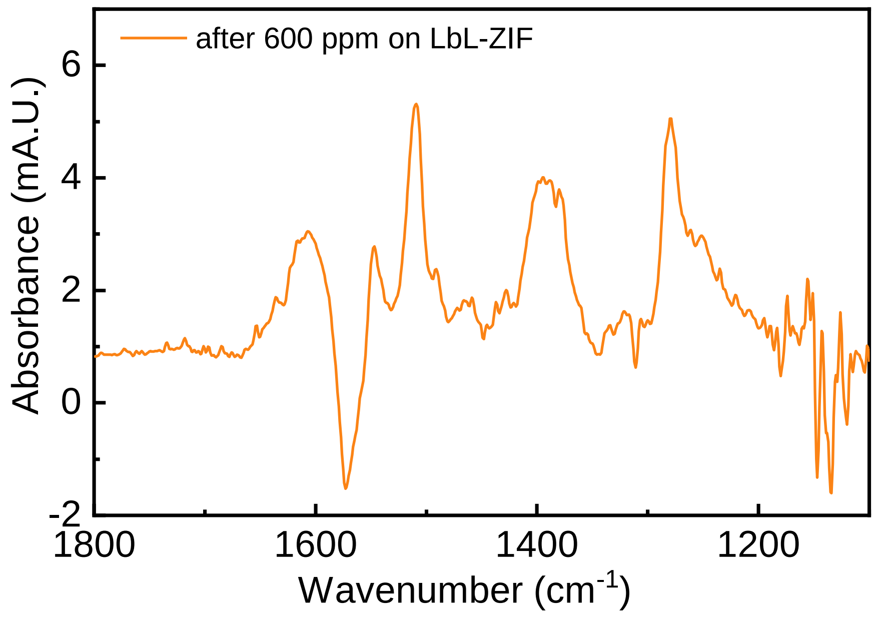

**Supplementary Figure 39**. FTIR-ATR spectra of FA adsorbed on ZIF-8 prepared by LbL. Background spectrum = $N_2$ flushed ZIF-8 film.

A control experiment with a blank Si ATR crystal was performed and the spectrum is shown in Supplementary Figure 40. No band associated with the observed FA species are visible.

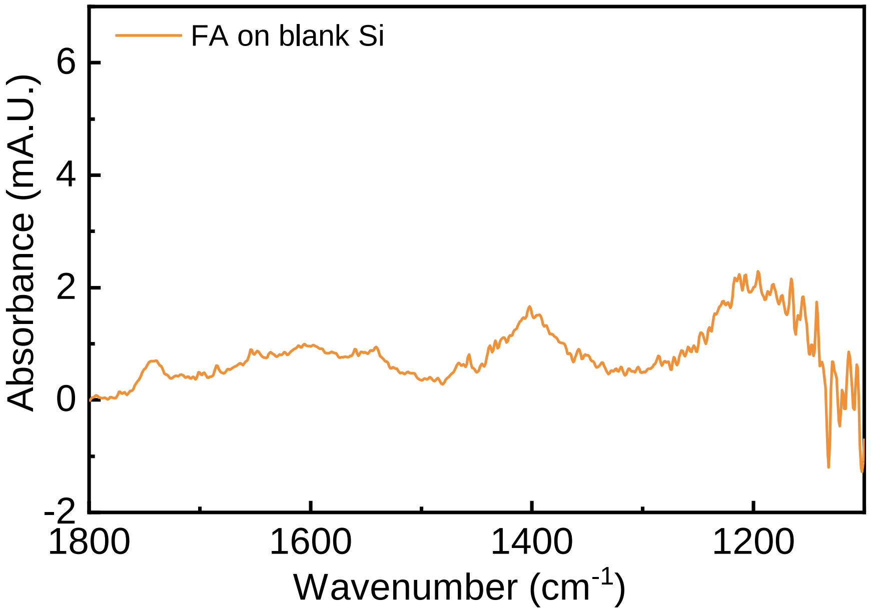


**Supplementary Figure 40**. FTIR-ATR spectra of 550 ppm FA adsorbed on blank Si ATR crystal. Background spectrum = Si ATR flushed with $N_2$.

All considered, the ATR-IR results prove that bands associated with all species observed using PiFM can be found in the bulk experiments. We observed that the same species found at lower FA concentrations in PiFM experiments are present in the ATR experiments, followed by an increase in concentration of formates. However, while PiFM allows to pick a ZIF-8 crystal or even a certain crystal plain for FA adsorption and thereby ensure a controlled defect-free experiment, the exclusion of defective ZIF-8 crystals cannot be avoided in bulk measurements. Therefore, PiFM gives access to adsorption studies on defined crystal planes beyond standard FTIR spectroscopy.

**Supplementary References**


1. Shekhah, O. *et al.* Step-by-step route for the synthesis of metal-organic frameworks. *J. Am. Chem. Soc.* **129**, 15118–15119 (2007).
2. Liu, Y. *et al.* TXM-Wizard: a program for advanced data collection and evaluation in full-field transmission X-ray microscopy. *J. Synchrotron Radiat.* **19**, 281–287 (2012).
3. Weng, T. & R. Schmidt, J. Flexible and Transferable ab Initio Force Field for Zeolitic Imidazolate Frameworks: ZIF-FF. *J. Phys. Chem. A* **123**, 3000–3012 (2019).
4. Chizallet, C. *et al.* Catalysis of Transesterification by a Nonfunctionalized Metal−Organic Framework: Acido-Basicity at the External Surface of ZIF-8 Probed by FTIR and ab Initio Calculations. *J. Am. Chem. Soc.* **132**, 12365–12377 (2010).
5. Kühne, T. D. *et al.* CP2K: An electronic structure and molecular dynamics software package - Quickstep: Efficient and accurate electronic structure calculations. *J. Chem. Phys.* **152**, 194103 (2020).
6. Perdew, J. P., Burke, K. & Ernzerhof, M. Generalized Gradient Approximation Made Simple. *Phys. Rev. Lett.* **77**, 3865–3868 (1996).
7. Grimme, S., Antony, J., Ehrlich, S. & Krieg, H. A consistent and accurate ab initio parametrization of density functional dispersion correction (DFT-D) for the 94 elements H-Pu. *J. Chem. Phys.* **132**, 154104 (2010).
8. Mathur, A., Sharma, P. & Cammarata, R. C. Negative surface energy — clearing up confusion. *Nat. Mater.* **4**, 186 (2005).
9. Łodziana, Z., Topsøe, N.-Y. & Nørskov, J. K. A negative surface energy for alumina. *Nat. Mater.* **3**, 289–293 (2004).
10. Treps, L., Gomez, A., de Bruin, T. & Chizallet, C. Environment, Stability and Acidity of External Surface Sites of Silicalite-1 and ZSM-5 Micro and Nano Slabs, Sheets, and Crystals. *ACS Catal.* **10**, 3297–3312 (2020).

11. Hu, Z. & Zhao, D. Metal–organic frameworks with Lewis acidity: synthesis, characterization, and catalytic applications. *CrystEngComm* **19**, 4066–4081 (2017).

12. Canivet, J., Vandichel, M. & Farrusseng, D. Origin of highly active metal–organic framework catalysts: defects? Defects! *Dalt. Trans.* **45**, 4090–4099 (2016).

13. A. Van Santen, R. Complementary Structure Sensitive and Insensitive Catalytic Relationships. *Acc. Chem. Res.* **42**, 57–66 (2008).

14. Somorjai, G. A. Active Sites in Heterogeneous Catalysis (eds. Eley, D. D., Pines, H. & Weisz) **26**, 1–68 (Academic Press, 1977).

15. Che, M. & Bennett, C. O. The Influence of Particle Size on the Catalytic Properties of Supported Metals. in (eds. Eley, D. D., Pines, H. & Weisz) **36**, 55–172 (Academic Press, 1989).

16. Boudart, M. Heterogeneous catalysis by metals. *J. Mol. Catal.* **30**, 27–38 (1985).

17. Bennett, C. O. & Che, M. Some geometric aspects of structure sensitivity. *J. Catal.* **120**, 293–302 (1989).

18. Vogt, C. *et al.* Understanding carbon dioxide activation and carbon–carbon coupling over nickel. *Nat. Commun.* **10**, 5330 (2019).

19. Vogt, C. *et al.* Unravelling structure sensitivity in CO2 hydrogenation over nickel. *Nat. Catal.* **1**, 127–134 (2018).

20. Van Hardeveld, R. & Van Montfoort, A. The influence of crystallite size on the adsorption of molecular nitrogen on nickel, palladium and platinum: An infrared and electron-microscopic study. *Surf. Sci.* **4**, 396–430 (1966).

21. Stakheev, A. Y., Mashkovskii, I. S., Baeva, G. N. & Telegina, N. S. Specific features of the catalytic behavior of supported palladium nanoparticles in heterogeneous catalytic reactions. *Russ. J. Gen. Chem.* **80**, 618–629 (2010).

22. Slater, A. G. & Cooper, A. I. Function-led design of new porous materials. *Science* **348**, aaa8075 (2015).

23. Derouane, E. G. Shape selectivity in catalysis by zeolites: The nest effect. *J. Catal.* **100**, 541–544 (1986).

24. Dwyer, F. G. Structure Sensitivity in Zeolite Catalysts. in *Structure-Activity and Selectivity Relationships in Heterogeneous Catalysis* (eds. Grasselli, R. K. & Sleight, A.) **67**, 179–192 (Elsevier, 1991).

25. Pérez-Ramírez, J., Kapteijn, F. & Brückner, A. Active site structure sensitivity in N2O conversion over FeMFI zeolites. *J. Catal.* **218**, 234–238 (2003).

26. Leblebici, S. Y. *et al.* Facet-dependent photovoltaic efficiency variations in single grains of hybrid halide perovskite. *Nat. Energy* **1**, 16093 (2016).

27. Li, X. *et al.* Three-dimensional vectorial imaging of surface phonon polaritons. *Science* **371**, 1364–1367 (2021).

28. Koper, M. T. M. Structure sensitivity and nanoscale effects in electrocatalysis. *Nanoscale* **3**, 2054–2073 (2011).

29. Ertl, G. Reactions at Surfaces: From Atoms to Complexity (Nobel Lecture). *Angew. Chem. Int. Ed.* **47**, 3524–3535 (2008).

30. Wolff, J., Papathanasiou, A. G., Kevrekidis, I. G., Rotermund, H. H. & Ertl, G. Spatiotemporal Addressing of Surface Activity. *Science* **294**, 134–137 (2001).

31. Somorjai, G. A. The experimental evidence of the role of surface restructuring during catalytic reactions. *Catal. Letters* **12**, 17–34 (1992).

32. Kim, M. *et al.* Controlling Chemical Turbulence by Global Delayed Feedback: Pattern Formation in Catalytic CO Oxidation on Pt(110). *Science* **292**, 1357–1360 (2001).

33. van Spronsen, M. A., Frenken, J. W. M. & Groot, I. M. N. Surface science under reaction conditions: CO oxidation on Pt and Pd model catalysts. *Chem. Soc. Rev.* **46**, 4347– 4374 (2017).

34. Zhang, Y. *et al.* Tuning reactivity of Fischer–Tropsch synthesis by regulating $TiO_x$ overlayer over Ru/TiO2 nanocatalysts. *Nat. Commun.* **11**, 3185 (2020).

35. van Santen, R. A., Ghouri, M. M., Shetty, S. & Hensen, E. M. H. Structure sensitivity of the Fischer–Tropsch reaction; molecular kinetics simulations. *Catal. Sci. Technol.* **1**, 891–911 (2011).

36. Roeffaers, M. B. J. *et al.* Spatially resolved observation of crystal-face-dependent catalysis by single turnover counting. *Nature* **439**, 572–575 (2006).

37. Ameloot, R. *et al.* Three-Dimensional Visualization of Defects Formed during the Synthesis of Metal–Organic Frameworks: A Fluorescence Microscopy Study. *Angew. Chem. Int. Ed.* **52**, 401–405 (2013).

38. Dong, S. & K. Dasgupta, P. Solubility of gaseous formaldehyde in liquid water and generation of trace standard gaseous formaldehyde. *Environ. Sci. Technol.* **20**, 637–640 (2002).

39. Maurer, G. Vapor-liquid equilibrium of formaldehyde-and water-containing multicomponent mixtures. *AIChE J.* **32**, 932–948 (1986).

40. Hou, C., Xu, Q., Peng, J., Ji, Z. & Hu, X. (110)-Oriented ZIF-8 Thin Films on ITO with Controllable Thickness. *ChemPhysChem* **14**, 140–144 (2013).

41. Chernikova, V., Shekhah, O. & Eddaoudi, M. Advanced Fabrication Method for the Preparation of MOF Thin Films: Liquid-Phase Epitaxy Approach Meets Spin Coating Method. *ACS Appl. Mater. Interfaces* **8**, 20459–20464 (2016).

42. Kida, K., Fujita, K., Shimada, T., Tanaka, S. & Miyake, Y. Layer-by-layer aqueous rapid synthesis of ZIF-8 films on a reactive surface. *Dalt. Trans.* **42**, 11128–11135 (2013).

43. Wagner, T. ij-particlesizer: ParticleSizer 1.0.1. (2016). doi:10.5281/ZENODO.56457

44. Baumgartner, B., Hayden, J., Lendl, B., Mesoporous silica films for sensing volatile organic compounds using attenuated total reflection spectroscopy. *Sensors Actuators B Chem.* **302**, 127194 (2020).

45. Baumgartner, B., Mashita, R., Fukatsu, A., Okada, K., Takahashi, M. Guest Alignment

and Defect Formation during Pore Filling in Metal–Organic Framework Films. *Angew. Chem. Int. Ed.* **61**, e202201725 (2022).

46. Yim, C. *et al.* Adsorption and desorption characteristics of alcohol vapors on a nanoporous ZIF-8 film investigated using silicon microcantilevers. *Chem. Commun.* **51,** 6168–6171 (2015).

47. Cousin Saint Remi, J. *et al.* Biobutanol Separation with the Metal–Organic Framework ZIF-8, *ChemSusChem*, **4**, 1074–1077 (2011).

48. Zhang, C., Gee, J.A., Sholl, D.S., Lively, R.P. Crystal-Size-Dependent Structural Transitions in Nanoporous Crystals: Adsorption-Induced Transitions in ZIF-8. *J. Phys. Chem. C*, **118**, 20727–20733 (2014)
